# c-JUN controls microbial colonization via selective phagocytosis in the sea anemone *Nematostella*

**DOI:** 10.1038/s41467-026-75511-w

**Published:** 2026-07-10

**Authors:** N. H. Kaya, M. Abukhalaf, G. Fuentes, J. Taubenheim, U. Hentschel, A. Tholey, S. Fraune

**Affiliations:** 1 Institute for Zoology and Organismic Interactions, Heinrich-Heine University Düsseldorf, Düsseldorf, Germany; 2 Systematic Proteome Research & Bioanalytics, Institute for Experimental Medicine, Christian-Albrechts-Universität zu Kiel, Kiel, Germany; 3 Research Unit Marine Symbioses, GEOMAR Helmholtz Centre for Ocean Research Kiel, Kiel, Germany; 4 Present Address: Research Group Medical Systems Biology, Institute for Experimental Medicine, Kiel University and University Hospital Schleswig-Holstein, Kiel, Germany

**Keywords:** Innate immunity, Microbiome

## Abstract

Innate immunity is traditionally viewed as a broad defense system with limited specificity. However, increasing evidence suggests that innate immune cells can discriminate between distinct microbial partners. How such specificity arises in early-diverging animals remains unclear. Here, we identify in the sea anemone *Nematostella vectensis* a selective host innate immune mechanism mediated by nematosomes, motile multicellular bodies that differentially process bacterial cells. Nematosomes preferentially engulf non-native *Vibrio* isolates while showing reduced uptake of native host-associated strains. We identify the transcription factor cJUN as a key regulator of this process. CRISPR/Cas9-mediated knockout of *cJUN* reduces nematosome abundance, impairs lysosomal response, alters microbiome assembly, and increases susceptibility to bacterial infection. These results link immune gene function to microbial selectivity and demonstrate that even early-diverging animals exhibit sophisticated innate immunity mechanisms for microbiome regulation. Our findings support the idea that immune specificity can arise through repurposing deeply conserved pathways and may have deep evolutionary origin.

## Abstract (web summary)

Here, Kaya *et al*. show that the transcription factor c-JUN enables selective phagocytosis of microbes in the sea anemone Nematostella vectensis, helping to shape its microbiome and thereby revealing an ancient mechanism of innate immune regulation.

## Introduction

The maintenance of a healthy functioning metaorganism, consisting of the host and its associated microorganisms, relies on an efficient host immune system. The immune system is based on a complex interplay of mechanisms protecting the host from pathogenic microorganisms, like bacteria^1,2^, fungi^3^ and viruses^4^, while simultaneously maintaining beneficial microbial relationships^5,6^. As part of the innate immune system, phagocytosis is a conserved mechanism found in invertebrates and vertebrates. Most likely, it evolved as a mechanism of nutrient uptake in unicellular eukaryotes^7^ and evolved during metazoan evolution^8^ to a mechanism of specialized immune cells to eliminate pathogens. Thereby, phagocytosis of foreign particles consists of target recognition, internalization into the phagosome and digestion of targets via phagolysosome formation^9–13^. While the cellular mechanisms of phagocytosis in invertebrates are similar to those in vertebrates^14^, the evolutionary conservation of specific gene functions remains to be determined^10^.

The widespread assumption is that the innate immunity provides an immediate, non-specific defense, while the adaptive immunity enables a targeted, antigen-specific response with an immunological memory^15^. However, recent research has challenged the traditional view that invertebrate immune systems lack specificity. Studies have shown that phagocytosis in invertebrates can exhibit a high degree of specificity^9,16^. In woodlice, hemocytes demonstrated increased phagocytosis of previously encountered bacterial strains, suggesting the ability to differentiate between closely related bacteria. In the squid-Vibrio symbiosis, hemocytes are able to differentiate between the squid’s preferred bacterial symbiont *Vibrio fisheri* and other bacteria of the *Vibrio* genus^17,18^. In addition, in a number of host-microbe interactions, it has been shown that phagocytes can both shape the microbiota and be influenced by specific members of the microbiome. In tse-tse flies, for example, hemocyte proliferation depends on colonization by *Wigglesworthia*^19^. A similar effect is observed in pea aphids, where the presence of some symbionts affects hemocyte abundance and the proportion of granulocytes in the hemocyte population^20^. We therefore hypothesize that mechanisms enabling microbial discrimination by innate immune cells may have evolved early in animal evolution, allowing ancestral animals to distinguish among different microorganisms.

In the following, we used the sea anemone *Nematostella vectensis* (*N. vectensis*) to study the specificity of phagocytosis and its effects on interactions with its microbiome in an early-branching metazoan. *N. vectensis* belongs to the cnidarians, a sister group of Bilateria, and exhibits a high genetic complexity characterized by similar signaling pathways for development and innate immunity compared to bilaterian animals^21,22^. The microbiome of *N. vectensis* shows a specific succession during host development and robust adaptations to environmental variations^23^, with a remarkable genotype-environment interaction influencing the variability of the microbiome^24,25^. The microbiome-mediated plasticity has been functionally linked to thermal adaptation in *N. vectensis*^25^.

**Fig. 1:**
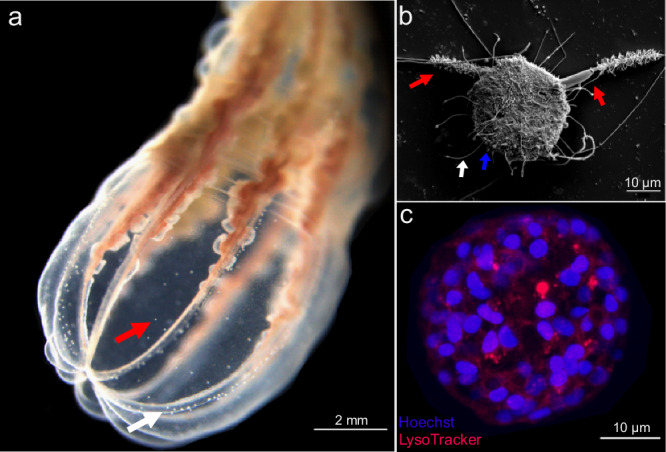
Nematosomes inside the polyp. **a** Foot region of adult N. vectensis polyp with nematosomes moving inside the body cavity (red arrows) and nematosomes resting on the body wall of the polyp (white arrows). Scalebar represents 2 mm. **b** SEM image of a single nematosome with two extending cnidocytes and discharged tubules (red arrows) and cilia highlighted with a blue arrow (Type 1) and with a white arrow (Type 2)^27^. The scalebar represents 10 µm. **c** Confocal image of a nematosome stained with Hoechst (blue), labeling nuclei, and LysoTracker (red), labeling acidic lysosomal compartments. The scalebar represents 10 µm. Representative images from 20 independent biological preparations with similar results are shown.

Microbiome establishment is strongly influenced by host regulatory mechanisms, with transcriptomic evidence indicating phagocytosis as a central process controlling early colonization events^26^. In *N. vectensis*, nematosomes (Fig. 1a), small motile multicellular bodies in the gastric cavity, are capable of phagocytosis and may act as immune bodies^27,28^. They originate from cnidoglandular tracts, the mesenteries, and consist of nematocytes and a second, not well characterized cell type, that shows phagocytic activity^27^.

While nematosomes can immobilize prey with the help of their nematocytes (Fig. 1b), the second cell type presumably serves the phagocytosis of bacteria (Fig. 1c), indicating a potential dual function in nutrition and immunity^27,28^. In addition, nematosomes are equipped with two types of cilia surrounding the cell complex^27^ (Fig. 1b), which facilitate their active mobility within the fluid of the gastric cavity (Supplementary Movie 1). A further indication of the active contribution to immunity is the fact that nematosomes co-express components of the TLR signaling pathway, such as TLR and NF-κB^29^. During oogenesis, the nematosomes are packed into the egg clutches by the female polyps, which are then expelled by the female polyps with a gelatinous matrix mucus (Supplementary Fig. 1a, b). However, there is yet to be any direct evidence that nematosomes are involved in the host immune response or that they can differentiate bacteria at the level of phagocytosis. In addition, the molecular regulation of nematosome function remains largely unknown. Previous transcriptome analyses have shown that a cJUN ortholog in *N. vectensis* responds to bacterial recolonization^26^, making it a promising candidate for regulating nematosome function (Supplementary Fig. 2). cJUN is an evolutionarily conserved transcription factor that activates immune genes, regulates phagocytosis, and is involved in cell proliferation^30–32^. As a central hub, cJUN integrates signaling information of various pathways, including ERK and JNK signaling^33^. In the cnidarian *Hydra,* TLR signaling via MyD88 activates JNK signaling following immune stimulation^34^, and in vertebrates, cJUN regulates macrophage activation^35,36^.

Here, we show that nematosomes exhibit selective phagocytosis, efficiently ingesting foreign *Vibrio* isolates and degrading them in the lysosome while sparing native *Vibrio* isolates. This selective phagocytosis correlates with the bacteria's ability to colonize *N. vectensis* adult polyps. Proteomic analyses revealed distinct protein enrichment patterns linked to a phagosomal pathway in response to foreign bacteria, highlighting cJUN’s role in immune-related trafficking. CRISPR/Cas9-mediated *cJUN* knockout (*cJUN*^−/−^) resulted in a significant reduction in nematosome abundance and impaired lysosomal activation after engulfment of *Vibrio* cells. These *cJUN*^−/−^ polyps exhibit a reduced fitness after *Vibrio coralliilyticus* (*V. coralliilyticus*^37^) infection and are colonized by an altered microbiome, demonstrating a causal relationship between the selective phagocytosis of the nematosomes and microbiome colonization. Our data provide strong evidence that cJUN is a key regulator of nematosome abundance and lysosome maturation in *N. vectensis*, playing a central role in microbiome regulation via phagocytosis.

## Results

### Nematosomes phagocytose *Vibrio* strains with varying efficiency

**Fig. 2:**
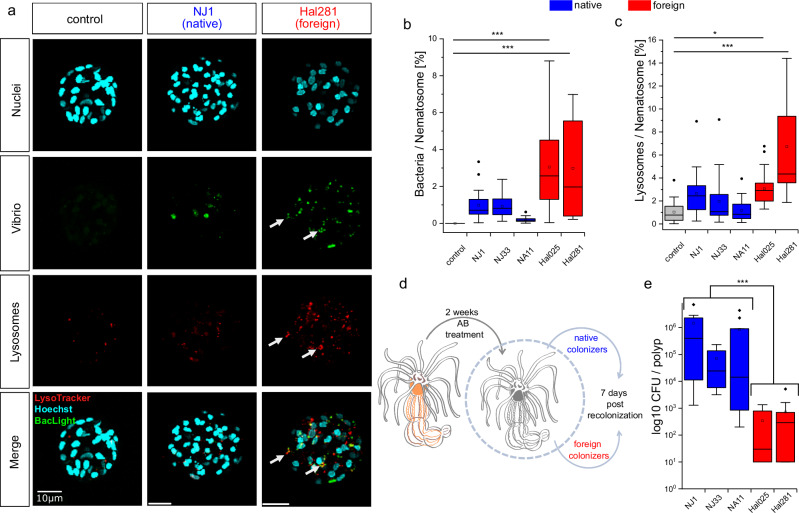
Nematosomes selectively phagocytose different Vibrio strains. **a** Representative confocal images of nematosomes challenged with NJ1 (native colonizer), Hal281 (foreign colonizer) and no bacterial challenge (control), lysosomes (red, LysoTracker), nuclei (blue, Hoechst), and Vibrios (green, BacLight). White arrows indicate the overlay of bacterial and lysosomal signal, representing bacterial degradation. Scalebars represent 10 µm. **b** Quantification of bacterial signal relative to total nematosome area (%). Differences among bacterial engulfment were assessed using a Kruskal-Wallis test (χ² (5) = 80.73, *p* < 0.0001) followed by Dunn’s multiple comparison test. *N* = 21 (control, NJ1, NJ33), *N* = 20 (NA11, Hal025), and *N* = 10 (Hal281) biologically independent nematosomes, * *p* <= 0.05, ** *p* <= 0.01, *** *p* <= 0.001. Box plots indicate median (middle line), interquartile range (box), and range (whiskers), as well as outliers (single points). **c** LysosTracker-positive lysosomal area relative to total nematosome area (%). Differences among treatments were assessed using Kruskal-Wallis test (χ² (5) = 43.49, *p* < 0.0001) followed by Dunn’s multiple comparison test. *N* = 21 (control, NJ1, NJ33, Hal025), *N* = 20 (NA11), and *N* = 11 (Hal281) biologically independent nematosomes, * *p* <= 0.05, ** *p* <= 0.01, *** *p* <= 0.001. Box plots indicate median (middle line), interquartile range (box), and range (whiskers), as well as outliers (single points). **d** Schematic representation of the experimental setup. Adult N. vectensis polyps underwent a two-week antibiotic (AB) treatment to deplete resident microbiota, followed by mono-association with either native or foreign Vibrio strains. Colonization was assessed after seven days post-recolonization (dpr). **e** Quantification of Vibrio colonization levels in adult polyps 7 dpr. *N* = 9 (NJ1, NA11), *N* = 4 (NJ33), *N* = 11 (Hal025), and *N* = 13 (Hal281) biologically independent polyps, two-way ANOVA revealed significant differences between the groups native (blue) and foreign (red) isolates, F _(1, 36)_ = 57.19, *p* = 6.095 × 10^-9^. * *p* <= 0.05, ** *p* <= 0.01, *** *p* <= 0.001. Box plots indicate median (middle line), interquartile range (box), and range (whiskers), as well as outliers (single points).

To test if nematosomes were phagocytosing bacteria differentially, we established a phagocytosis assay that allowed us to quantify, on one hand, lysosomal activity and, on the other hand, bacterial engulfment (Fig. 2a).

Therefore, nematosomes were extracted from the gastric cavity and incubated in a bacterial suspension with a defined bacterial concentration. For the assay, we chose different *Vibrio* strains, as they are common marine bacteria and were among the main colonizers of *N. vectensis*^23,24^. As native isolates, we selected the *Vibrio* isolates NJ1, NJ33 and NA11 that were cultivated from *N. vectensis* (Supplementary Table 1)^26^. We analyzed the phagocytosis rate of these native isolates and compared it with the rate of phagocytosis of foreign *Vibrio* isolates (Hal025 and Hal281^38^) derived from the sponge *Halichondria panicea* (Supplementary Table 1). Native *Vibrio* strains were phagocytosed at significantly lower rates, while the foreign isolates Hal025 and Hal281 were engulfed at substantially higher rates (Fig. 2a, b). The increased phagocytosis of foreign isolates correlated with a significant increase in lysosomal activity within nematosomes (Fig. 2a, c). In contrast, nematosomes confronted with native *Vibrio* strains did not increase their lysosomal activity (Fig. 2a, c).

In addition to the phagocytosis rate for each isolate, we also compared the colonization efficiency of each isolate in mono-association experiments (Fig. 2d, e). This recolonization approach revealed that the three native *Vibrio’s*, namely NJ1, NJ33 and NA11, colonized in significantly higher rates on the polyp 7 days post recolonization (dpr), compared to the two foreign isolates Hal025 and Hal281 (Fig. 2e). This trend was already seen after 2 dpr (Supplementary Fig. 4). These results correlate with the observations of elevated phagocytosis rates (Fig. 2b) and lysosomal activity (Fig. 2c) of the nematosomes upon foreign *Vibrio* engulfment suggesting a potential link between nematosome phagocytosis and colonization success.

### Foreign and native *Vibrio* isolates cause diverging proteome responses in nematosomes

**Fig. 3:**
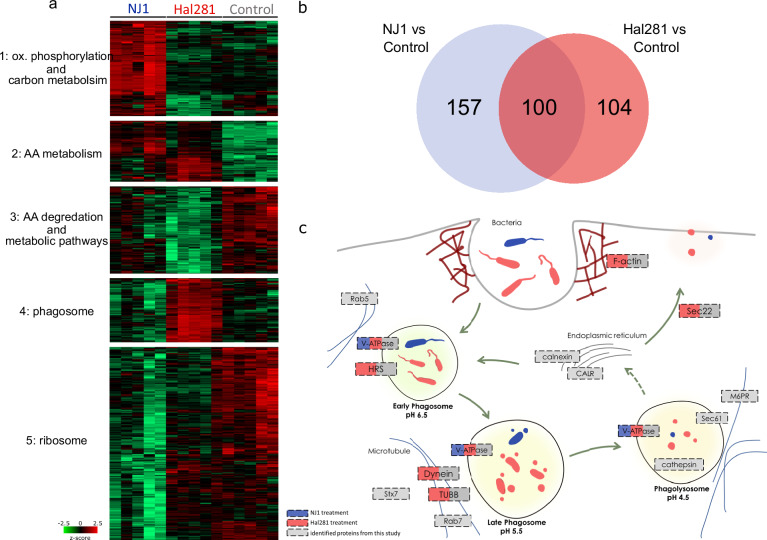
Differentially abundant proteins in native vs foreign treatment in nematosomes. This study was conducted with five biological replicates per group: a control group without bacterial treatment, a group treated with a native isolate (NJ1) and a group treated with a foreign isolate (Hal281). A total of 2676 proteins were detected, of which 257, 204 and 77 proteins showed increased abundance in the native isolate-treated group, the foreign isolate-treated group and the untreated control group, respectively. **a** heatmap showing clustering of differentially abundant proteins across three conditions: nematosomes treated with NJ1, Hal281, and untreated control. Five distinct protein clusters are identified based on functional enrichment. Protein abundance is represented as Z-scores, with red indicating higher and green lower abundance. (Permutation-based FDR ANOVA, *p* < 0.01, *N* = 826). **b** Venn diagram illustrating the proteins identified uniquely in NJ-treated (157 proteins) and uniquely in Hal281-treated (104 proteins), and 100 proteins shared between NJ1 and Hal281 treatments. **c** Schematic representation of the phagosome-lysosome pathway in nematosomes following bacterial exposure. The diagram shows the proposed trafficking routes from bacterial uptake, early phagosome formation, lysosomal fusion, and subsequent degradation^99–101^. Proteins significantly enriched in NJ1-treated (blue) and Hal281-treated (red) nematosomes are mapped onto corresponding pathway components, suggesting differential regulation of lysosomal processing depending on the bacterial isolate. Gray boxes represent proteins detected regarding phagosomes in this study.

To characterize the differential response of nematosomes to native and foreign *Vibrio* strains, we performed proteome analysis with extracted nematosomes. Specifically, we assessed the responses of nematosomes after confronting them with NJ1 and Hal281, and compared them to a control treatment without bacterial challenge. The proteome analysis revealed significantly abundant proteins when comparing nematosomes challenged with native and foreign bacterial isolates (Fig. 3, Supplementary Data 1, Supplementary Fig. 5). A total of 2,676 proteins were detected in the proteomic analysis of nematosomes treated with bacterial isolates NJ1 and Hal281, as well as in untreated controls. To extract proteins that were uniquely differently abundant in either NJ1 or Hal281 treatment, we generated five clusters using *k*-means clustering (Fig. 3a). Out of the total proteins identified, 157 proteins were detected uniquely in NJ1-treated samples and 104 in Hal281-treated nematosomes (Fig. 3b). Thereby, cluster 1 represented proteins which emerged exclusively following NJ1 treatment, while cluster 4 contain proteins, which were higher in abundance after confrontation with Hal281. A KEGG enrichment analysis revealed that cluster 1, contains proteins related to carbon and nitrogen metabolism (Supplementary Data 2), suggesting that interaction with native bacteria might promote the host metabolism. In contrast, the treatment with Hal281 elevated the abundance of proteins belonging to the phagosomal pathway (Supplementary Data 2, 3). Proteins belonging to the cytoskeleton formation of phagosomal formation, like Dynein and Tubulin beta (TUBB) as well as F-actin were especially increased (Fig. 3c, Supplementary Data 3). Interestingly, the V-ATPase showed an increase in abundance in both bacterial treatments, potentially linking it to default lysosomal activity (Fig. 2c).

In summary, the proteomic analysis revealed elevated abundance in the phagosomal pathway upon foreign bacterial treatment, indicating an active immune response in nematosomes, particularly in recognizing and degrading foreign isolates. To further explore the molecular mechanisms driving these responses, we aimed to manipulate the regulatory pathways involved.

### *cJUN* mutation regulates host resistance to bacterial infection and reduces nematosome abundance

**Fig. 4:**
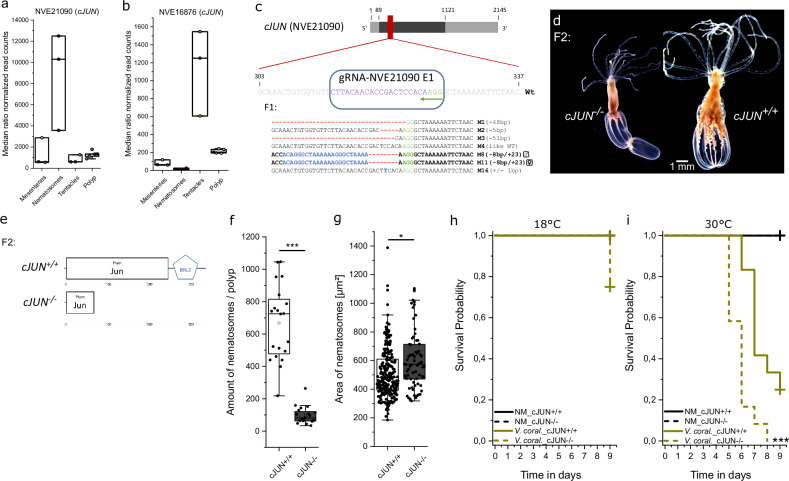
NVE21090 mutation alters nematosome abundance and increases susceptibility to bacterial infection. **a**,**b** NVE21090 (cJUN) and NVE16876 (cJUN) expression based on data from^26,27^. Median ratio normalized read counts are represented, showing tissue-specific expression patterns. *N* = 3 (Mesenteries, Nematosomes, Tentacles), *N* = 5 (whole Polyps). Box plots indicate median (middle line), interquartile range (box), and single points representing single biological replicates. **c** Schematic illustration of NVE21090 (cJUN) locus with guide recognition sites (UTR in bright gray and exon in dark gray). The F1 generation displays a range of mutations, including deletions (red), insertions (blue), and the PAM sequence (green). The guide sequence is highlighted in purple. **d** F2 adult polyps. Left cJUN^−/−^ and right cJUN^+/+^. Scalebar represents 1 mm. **e** Scheme of protein structure of wildtype and cJUN mutant F2 animals with essential domains missing in cJUN^−/−^ (generated with SMART). **f** Amount of nematosomes extracted from biologically independent cJUN^+/+^ and cJUN^−/−^ animals. N = 20 polyps / genotype, two-sided unpaired Student’s *t*-test: *t* = 10.68, df = 38, *p* < 0.0001***. Box plots indicate median (middle line), interquartile range (box), and range (whiskers). **g** Size of nematosomes measured by area of nematosomes in cJUN^+/+^ and cJUN^−/−^ polyps. *N* = 70 (cJUN^−/−)^ and *N* = 257 (cJUN^+/+^) biologically independent nematosomes, two-sided unpaired Student’s *t*-test, *t* = −2.51, df = 294, *p* = 0.013*. Box plots indicate median (middle line), interquartile range (box), and range (whiskers). **h**,**i** Kaplan-Me**i**er survival curves comparing the survival of cJUN^+/+^ and cJUN^−/−^ polyps under 18 °C (**h**) and 30 °C (**i**) infected with V. coralliilyticus or no bacterial infection (control group) observed for 9 days. Statistical differences between survival curves revealed significant differences among groups at 18 °C (log-rank test, χ² (3) = 9.40, *p* = 0.0244); pairwise comparison between cJUN^+/+^ and cJUN^−/−^ animals under V. coralliilyticus challenge did not reach statistical significance (*p* = 0.069). *N* = 12 / genotype (**h**). **i** Survival d**i**fferences among treatments at 30 °C were analyzed using log-rank test (χ² (3) = 55.67, *p* < 0.0001***). *N* = 12 / genotype.

In the next step, we aimed to alter the function of nematosomes by CRISPR/Cas9 genome editing. We identified a *cJUN* ortholog, which is highly expressed in nematosomes^27^ (NVE21090) and responded to bacterial recolonization^26^ (Fig. 4a and Supplementary Fig. 2a), while a second ortholog is mainly expressed in the tentacle region (NVE16876) (Fig. 4b) of the polyp and showed no change in expression after bacterial recolonization^26,39^ (Supplementary Fig. 2b).

Therefore, we selected the *cJUN* ortholog NVE21090 for CRISPR/Cas9 genome editing^40^, to investigate the function of nematosomes in host immunity and regulation of microbiome composition. We generated deletions in the first exon of NVE21090, which ultimately led to a variation of mutations in animals of the F1 generations (Fig. 4c). For the subsequent breeding, we selected the male strain M8 and the female strain M11, both carrying the same heterozygous mutation leading to a stop codon in the first exon of the gene., leading to incomplete translation and loss of essential domains within the protein (Fig. 4e). All subsequent approaches were conducted on F2 polyps with homozygous mutation named *cJUN*^−/−^.

Morphological comparisons between *cJUN*^*+/+*^ and *cJUN*^−/−^ polyps revealed no obvious differences in overall polyp morphology (Fig. 4d). However, *cJUN*^−/−^ polyps contained significantly fewer nematosomes compared to *cJUN*^*+/+*^ animals (Fig. 4f). While adult *cJUN*^*+/+*^ polyps harbored approximately 700 nematosomes, the same-sized *cJUN*^−/−^ polyps contained only around 100 nematosomes per polyp. Interestingly, this lower number seemed to be slightly compensated by size, as *cJUN*^−/−^ nematosomes displayed a bigger size compared to *cJUN*^*+/+*^ nematosomes (Fig. 4g).

Since nematosomes are maternally deposited into the egg packages, we next asked whether the cJUN mutation also affects the abundance of nematosomes transferred to developing embryos^27^ and the fitness of the corresponding offspring. Examination of egg packages released from adult female polyps revealed the presence of numerous nematosomes embedded within the gelatinous matrix surrounding the embryos (Supplementary Fig. 1a–c). Consistent with the reduced nematosomes abundance observed in female adult mutants, egg packages derived from *cJUN*^−/−^ animals contained significantly fewer nematosomes compared to those from *cJUN*^*+/+*^ female animals (Supplementary Fig. 1d, e). To assess the functional consequences of reduced maternal nematosome transfer, we monitored larval survival over an extended developmental period (Supplementary Fig. 1f). Offspring originated from *cJUN*^−/−^ female polyps exhibited a significant higher decline in survival over time, compared to offspring from *cJUN*^*+/+*^ mothers (Supplementary Fig. 1f). The survival rate of the offspring depended on the mother’s genotype and not on the offspring’s genotype, since *cJUN*^*+/+*^ oocytes fertilized with *cJUN*^−/−^ sperm, which produced also heterozygous F1 offspring, exhibit a similarly high survival rate as the offspring from a *cJUN*^*+/+*^ control crossing (Supplementary Fig. 1f). These findings suggest that maternal provisioning of nematosomes contributes to long-term offspring survival and early-life protection.

To determine whether the reduced nematosome abundance in *cJUN*^−/−^ animals affected host resilience to infection, we compared the survival of *cJUN*^*+/+*^ and *cJUN*^−/−^ polyps following exposure to the pathogenic bacterium *V. coralliilyticus* (Fig. 4h, i). Survival was monitored over nine days at two temperatures, 18 °C (Fig. 4h) and 30 °C (Fig. 4i), representing elevated stress conditions. Kaplan-Meier survival analysis revealed significant genotype-dependent differences in survival following pathogen exposure. While *cJUN*^*+/+*^ polyps showed higher survival rates during infection, *cJUN*^−/−^ animals exhibited a significantly increased mortality. This effect became particularly pronounced under elevated temperature conditions, where pathogen-induced mortality progressed more rapidly in mutant animals compared to *cJUN*^*+/+*^ polyps (Fig. 4i). In contrast, *cJUN*^−/−^ animals without bacterial challenge, as well as animals exposed to native and foreign colonizers, showed unaffected survival rates (Supplementary Fig. 1g, h). Interestingly, GF animals, independent of genotype, exhibit higher mortality following infection with *V. coralliilyticus* than animals with an intact microbiome (Supplementary Fig. 1i, j), suggesting that the microbiome provides general protection against infection with *V. coralliilyticus*. These findings indicate that cJUN contributes to host defense against bacterial infection and suggest that the reduced nematosome abundance in *cJUN*^−/−^ animals compromises the host’s ability to control pathogenic bacteria. To understand how *cJUN* mutation affects bacterial processing by nematosomes, we next examined the phagocytic response of mutant nematosomes toward different *Vibrio* strains.

### Loss of cJUN abolishes selective bacterial recognition by nematosomes

**Fig. 5:**
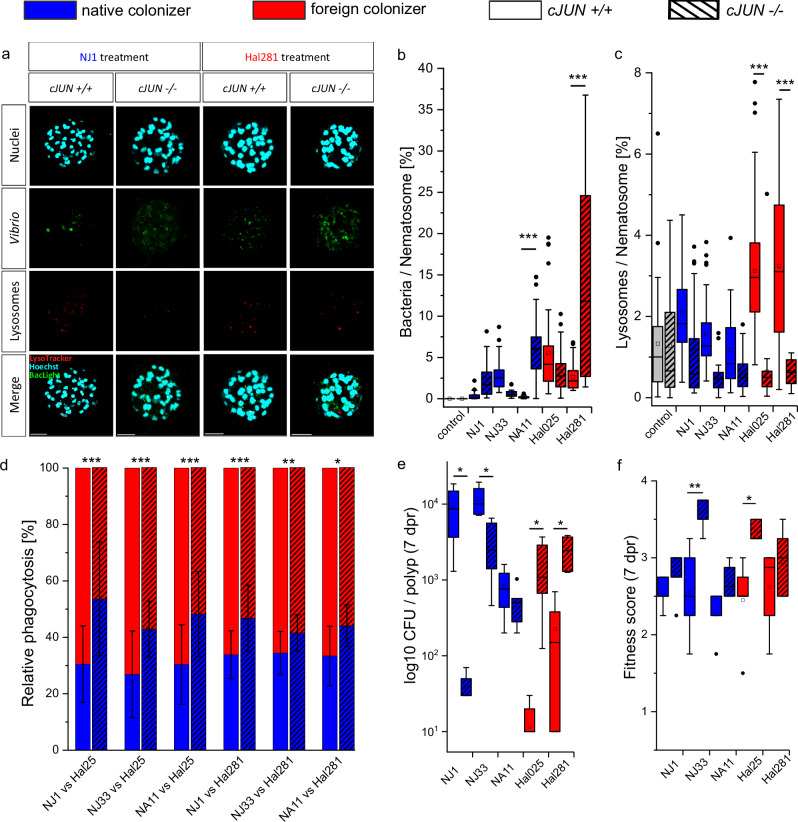
cJUN is required for selective bacterial recognition by nematosomes. **a** Representative confocal images of cJUN-depleted and not depleted nematosomes challenged with NJ1 or Hal281, lysosomes (red, LysoTracker), nuclei (blue, Hoechst), and Vibrios (green, BacLight). Scalebars represent 10 µm. **b** Quantification of bacterial signal relative to total nematosome area (%). N (cJUN^+/+^) = 26 (control), 32 (NJ1), 35 (NJ33), 20 (NA11), 43 (Hal025), 36 (Hal281), and N (cJUN^−/−^) = 17 (control), 22 (NJ1), 17 (NJ33), 41 (NA11), 26 (Hal025), 15 (Hal281) biologically independent nematosomes, Kruskal-Wallis ANOVA (χ² (11) = 228.72, *p* < 0.0001) followed by a Dunn’ multiple comparison, * *p* <= 0.05, ** *p* <= 0.01, *** *p* <= 0.001. Box plots indicate median (middle line), interquartile range (box), and range (whiskers), as well as outliers (single points). **c** LysoTracker-positive lysosomal area relative to total nematosome area (%) in response to different Vibrio isolates. N (cJUN^+/+^) = 26 (control), 33 (NJ1), 35 (NJ33), 20 (NA11), 43 (Hal025), 36 (Hal281), and N (cJUN^−/−^) = 33 (control), 21 (NJ1), 18 (NJ33), 41 (NA11), 26 (Hal025), 15 (Hal281) biologically independent nematosomes, Kruskal-Wallis ANOVA (χ² (11) = 152.22, *p* < 0.0001) followed by a Dunn’s multiple comparison, * *p* <= 0.05, ** *p* <= 0.01, *** *p* <= 0.001. Box plots indicate median (middle line), interquartile range (box), and range (whiskers), as well as outliers (single points). **d** Relative bacterial engulfment in nematosomes after bacterial challenges with native (blue) and foreign (red) Vibrio strains. Stack bars represent the proportion of engulfed bacteria relative to the total area of engulfed bacteria in cJUN^+/+^ and cJUN^−/−^ nematosomes. Statistical differences between genotypes were assessed using a two-sided Mann-Whitney U test. *N* = 27–53 nematosomes, * *p* <= 0.05, ** *p* <= 0.01, *** *p* <= 0.001. Stacked bars represent mean relative contribution of each isolate; error bars indicate ± s.d. across biological replicates. **e** Mono-association of native and foreign colonizers on adult polyps after 7 days post recolonization. Differences among th**e** isolates and genotypes were assessed using a Kruskal-Wallis test (χ² (9) = 40.86, *p* < 0.0001) followed by Dunn’s multiple comparison. N (cJUN^+/+^) = 4 (NJ1, NJ33, NA11), 6 (Hal025), 8 (Hal281), and N (cJUN^−/−^) = 4 (NJ1, Hal281), 5 (NJ33, NA11), 8 (Hal025) biologically independent polyps, * *p* <= 0.05, ** *p* <= 0.01, *** *p* <= 0.001. Box plots indicate median (middle line), interquartile range (box), and range (whiskers), as well as outliers (single points). **f** Polyp fitness scores at 7 days post recolonization (dpr) mono-association. Statistical significance was assessed using two-way ANOVA with genotype and treatment as **f** actors followed with Bonferroni multiple comparisons. Significant effects were detected for genotype (F _(1, 38)_ = 25.33, *p* < 0.0001) and treatment (F _(4, 38)_ = 2.89, *p* = 0.0348), whereas the interaction was not significant (F _(4, 38)_ = 2.05, *p* = 0.107). *N* = 5 biologically independent polyps per treatment (*N* = 4 (cJUN^−/−^ NA11, cJUN^+/+^ Hal281), * *p* <= 0.05, ** *p* <= 0.01, *** *p* <= 0.001. Box plots indicate median (middle line), interquartile range (box), and range (whiskers), as well as outliers (single points).

To approach the effect of *cJUN* on nematosome phagocytosis, we confronted *cJUN*^−/−^ and *cJUN*^*+/+*^ polyps with the same bacterial isolates (Fig. 5a). *cJUN*^*+/+*^ revealed similar lysosomal activity and rates of phagocytosis as observed in WT animals after confrontation with foreign and native *Vibrio* strains (Fig. 2b, c and Fig. 5a–c). However, while nematosomes adjusted their lysosomal activity in response to different isolates, *cJUN*^−/−^ nematosomes maintained a low lysosomal activation independent of bacterial treatment (Fig. 5c). Analyzing the number of bacteria engulfed in nematosomes revealed an increase in the isolates NA11 and Hal281 (Fig. 5b) in *cJUN*^−/−^ nematosomes, most likely by an accumulation of bacterial cells in the phagosome. Because the lysosome was not activated in *cJUN*−/− nematosomes, bacterial degradation in the phagosome was most likely impaired, resulting in arrested phagocytosis.

To determine whether the difference in lysosomal activation by foreign and native *Vibrio* isolates leads to selective phagocytosis, we conducted a phagocytosis competition experiment in which we exposed nematosomes to both a native and a foreign isolate at the same concentration (Fig. 5d). This experiment revealed that *cJUN*^*+/+*^ nematosomes preferentially phagocytose foreign isolates at a phagocytosis rate of approximately 70%, whereas mutant nematosomes lose this preferential phagocytosis of foreign isolates and engulf both bacteria at a similar rate (Fig. 5d).

The impaired selective phagocytosis of foreign *Vibrio* isolates resulted in an increased recolonization rate in mono-association experiments (Supplementary Fig. 6a) in adult *cJUN*^−/−^ polyps compared to *cJUN*^*+/+*^ polyps at 7dpr (Fig. 5e and Supplementary Fig. 6b). Interestingly, the native isolates NJ1 and NJ33 recolonized *cJUN*^−/−^ polyps significantly lower compared to *cJUN*^*+/+*^ polyps, suggesting that nematosomes may facilitate the establishment of specific native bacterial colonizers. To evaluate whether these altered colonization dynamics affect host health, we assessed polyp fitness scores following the bacterial mono-associations (Fig. 5f and Supplementary Figs. 3, 6c). The scoring revealed significant effects of both genotype and bacterial treatment, with mutant animals displaying reduced health under specific bacterial challenges compared to WT animals.

### Bacterial dysbiosis in *cJUN*^−/−^ polyps

Discovering significant differences in the phenotype of *cJUN*^−/−^ polyps, we proceeded to analyze its associated microbiome. 16S rRNA gene sequencing revealed significant differences between the microbiome of *cJUN*^−/−^ and *cJUN*^*+/+*^ polyps (Supplementary Fig. 7, Supplementary Table 2), with a significantly lower alpha diversity (Supplementary Fig. 7c) and evenness (Supplementary Fig. 7d) compared to *cJUN*^*+/+*^ animals.

**Fig. 6:**
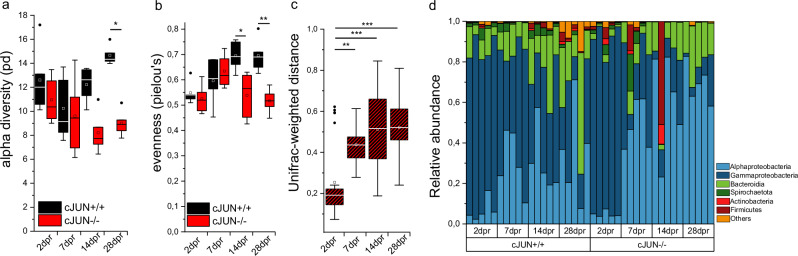
Microbiome comparison between cJUN^+/+^ and cJUN^−/−^ after recolonization. **a**,**b** Alpha diversity metrices for cJUN^+/+^ and cJUN^−/−^ microbiome comparison over a time course of 1 month (2, 7, 14, 28 dpr). **a** Faith’s phylogenetic diversity, reflecting the evolutionary richness of bacterial communities. **b** Pielou’s evenness, indicating how evenly bacterial taxa are distributed within each community. *N* = 5 / genotype, Kruskal-Wallis ANOVA (χ² (7) = 20.57, *p* = 0.0045) followed by Dunn’s multiple comparison test, * *p* <= 0.05, ** *p* <= 0.01, *** *p* <= 0.001. Box plots indicate median (middle line), interquartile range (box), and range (whiskers), as well as outliers (single points).**c** Weighted UniFrac distance comparisons between microbial communities of cJUN^+/+^ and cJUN^−/−^ polyps over time. Weighted UniFrac considers both bacterial relatedness and relative abundance, with larger distances indicating greater differences between microbial communities. *N* = 5 polyps for each dpr. Kruskal-Wallis ANOVA (χ² (7) = 22.23, *p* = 0.0023) followed by Dunn’s multiple comparison test, * *p* <= 0.05, ** *p* <= 0.01, *** *p* <= 0.001. Box plots indicate median (middle line), interquartile range (box), and range (whiskers), as well as outliers (single points). **d** Bar plots representing the relative abundance of bacterial taxa in cJUN^+/+^ and cJUN^−/−^ animals over time. Each bar shows the mean relative abundance of taxa across samples for each genotype, highlighting differences in the microbial community composition among the two groups and in the timepoints after recolonization.

These differences in bacterial colonization in *cJUN*^−/−^ and *cJUN*^*+/+*^ polyps support previous results indicating that host mechanisms are involved in the control of bacterial establishment in *N. vectensis*^24^. To test this hypothesis, we recolonized germfree adult *cJUN*^*+/+*^ and *cJUN*^−/−^ polyps with the bacterial consortia of adult polyps and followed the succession of bacterial establishment over the period of 28 days by 16S rRNA gene sequencing. Over the whole course of the experiment, the cJUN genotype had significant effects on microbial community structure (Table 1 and Fig. 6a–c), while the time point after recolonization (days post recolonization, dpr) accounted for differences in microbial colonization (Table 1).

**Table 1:** Statistical summary of ADONIS and ANOSIM tests on Bray-Curtis, Jaccard, Weighted UniFrac, and Unweighted UniFrac distance matrices, comparing microbial community dissimilarities between *cJUN*^*+/+*^ and *cJUN*^−/−^ animals

Parameter	Metric	Adonis R²		Adonis p		ANOSIM R		ANOSIM p
genotype	Bray-Curtis		0.097		0.005	0.17	0.002	
	Jaccard		0.099		<0.001	0.31	<0.001	
	Weighted UniFrac		0.073		0.031	0.14	0.007	
	Unweighted UniFrac		0.101		<0.001	0.23	<0.001	
dpr	Bray-Curtis		0.174		<0.001	0.42	<0.001	
	Jaccard		0.111		<0.001	0.40	<0.001	
	Weighted UniFrac		0.282		<0.001	0.49	<0.001	
	Unweighted UniFrac		0.110		<0.001	0.28	<0.001	

The parameter column indicates whether the analysis was performed at the genotype level or at the dpr level. Adonis R^2^ values represent the proportion of variance explained by genotype or dpr while ANOSIM R values indicate the degree of separation between groups, 999 permutations. Significant differences are indicated by *p*-values, with higher R values reflecting stronger microbial dissimilarities between groups.

During early time points (2 and 7 dpr) *cJUN*^*+/+*^ and *cJUN*^−/−^ polyps exhibited similar patterns in microbial richness and evenness (Fig. 6a, b). After 14 dpr, the microbiome of *cJUN*^*+/+*^ polyps showed significantly higher bacterial alpha diversity and evenness compared to *cJUN*^−/−^ polyps (Fig. 6a, b). The increase in Weighted UniFrac distances between the microbiome of the different genotypes revealed that genotype-related differences in the composition of the microbiome become more pronounced over time, as the microbial communities developed differently between *cJUN*^*+/+*^ and *cJUN*^−/−^ animals (Fig. 6c). The significant differences in specific microbial taxa observed between the genotypes over time (Fig. 6d and Supplementary Fig. 8), including the dominance of some Alphaproteobacteria taxa (Supplementary Fig. 8a, b) and the reduced colonization success of specific Gammaproteobacteria (Supplementary Fig. 8d) in *cJUN*^−/−^ polyps highlight the selective force of nematosomes on specific taxa.

## Discussion

### Nematosomes exhibit characteristics of ancient immune cells

Our study provides strong evidence that nematosomes are key immune structures in *N. vectensis*, playing a critical role in microbial selection through differential phagocytosis. The phagocytosis assays demonstrated that nematosomes exhibit a clear preference for engulfing foreign *Vibrio* isolates while sparing native host-associated ones, indicating that bacterial discrimination acts most likely on the level of recognition. Phagocytosis is a fundamental immune defense mechanism in invertebrates, often mediated by circulating cells called immunocytes. These cells fulfill various functions of the innate immune system, including the recognition of pathogens, phagocytosis and the synthesis of antimicrobial proteins. Thereby, the innate response triggered by microbial-associated molecular patterns (MAMPs) is based on the activation of pattern recognition receptors. In vertebrates, macrophages use pattern recognition receptors (PRRs), such as Toll-like receptors (TLRs) and scavenger receptors to recognize MAMPs and activate downstream immune pathways, including NF-κB^41,42^. In nematosomes of *N. vectensis* components of the TLR signaling pathway have previously been reported^29,43^. However, whether these pathways directly regulate nematosome-mediated phagocytosis remains unknown and was not tested in the present study. Experimental MyD88 knockdowns in *Hydra* showed that TLR signaling can also be linked to cJUN, resulting in altered microbiome composition and impaired bacterial recognition^34^.

The observed correlation between phagocytosis efficiency and bacterial colonization rate supports the hypothesis that nematosomes act as selective gatekeepers of microbial establishment in *N. vectensis*. This correlation was functionally linked in *cJUN*^−/−^ nematosomes that lost the ability to differentiate between foreign and native isolates in a competition phagocytosis assay. Native *Vibrio* isolates were phagocytosed at the same rate as foreign isolates in *cJUN*^−/−^ polyps, in contrast to lower phagocytosis rates in WT polyps. In addition, colonization by foreign isolates increased in *cJUN*^−/−^ polyps, suggesting that nematosomes likely help to shape the microbiome by selectively removing bacteria before they become stably established in the host. The general immunological character of nematosomes was also evident in an infection experiment using the pathogen *V. coralliilyticus*, in which cJUN^−/−^ polyps exhibited reduced fitness. The proteomic analysis further supports this immune-related function, revealing distinct protein patterns in nematosomes upon exposure to bacteria. Proteins involved in phagocytosis, including cytoskeletal components such as actin and dynein, were more abundant in response to foreign bacteria, supporting the notion that nematosomes are actively involved in immune surveillance and microbial selection and emphasizing their functional importance in host immunity.

### cJUN - a regulator of nematosome phagocytosis and proliferation

Our results highlight cJUN as a crucial regulator of innate immunity in *N. vectensis*. CRISPR/Cas9-mediated knockout of *cJUN* resulted in an impaired ability to mount a selective immune response. In *cJUN*^−/−^ mutants, nematosomes exhibited a failure to activate lysosomal degradation pathways following bacterial engulfment, leading to an accumulation of both native and foreign bacteria within the phagosome. Similarly, macrophages, disrupted in cJUN, TLRs, or NF-κB, can still engulf pathogens but fail to complete their degradation^44–46^, resulting in “arrested phagocytosis”, where bacteria remain trapped within the phagosomes but are not effectively digested. Certain intracellular pathogens, exploit these host weaknesses by preventing phagosome-lysosome fusion or modifying host signaling pathways to survive within immune cells^47–49^. These evasion tactics allow pathogens to manipulate key stages of the phagocytosis process, including phagosome formation, maturation, and acidification^49^. By interfering with these crucial immune defense mechanisms, bacteria can avoid degradation and establish intracellular infections. However, symbionts may also rely on the mechanism of arrested phagocytosis to persist within host tissue. In sponges, ankyrin-repeat proteins from bacterial symbionts can modulate phagocytosis by interfering with phagosome development, potentially allowing symbionts to escape digestion^50,51^. Similarly, in deep-sea mussels, the regulation of mTORC1 signaling helps retain symbionts in gill cells by preventing phagosome digestion^52^. In coral-dinoflagellate symbiosis, the symbiosome is hypothesized to be an early arrested phagosome, with transient gene expression changes occurring during symbiont uptake^53^. Recent results indicate that anthozoan hosts indiscriminately phagocytose various microalgae, but non-symbiotic species are expelled through vomocytosis. Successful symbionts suppress the host’s innate immune response, preventing expulsion and promoting niche formation^54^. These studies highlight the importance of phagocytic arrest in various symbiotic relationships among marine organisms.

In *N. vectensis cJUN*^−/−^ nematosomes exhibited a similar phenotype. They successfully internalized bacteria but failed to activate the lysosomal responses necessary for degradation. The accumulation of engulfed but undegraded bacteria in mutant nematosomes suggests that cJUN plays a crucial role in regulating lysosomal maturation and phagosomal acidification. This parallels its function described in vertebrates, where cJUN is involved in the transcriptional regulation of immune effectors, including lysosomal enzymes and phagosome maturation factors^35^. In *N. vectensis* the impaired cJUN-dependent signaling may thus reduce the degradation of foreign *Vibrio* strains, contributing to microbial persistence and altered colonization patterns. This suggests that cJUN is essential for orchestrating the cytoskeletal and phagosomal dynamics required for effective microbial clearance.

In addition, cJUN regulates nematosome abundance, as demonstrated by the significant reduction in nematosome numbers in *cJUN*^−/−^ mutants. It is well known that cJUN positively regulates cell proliferation by repressing tumor suppressor genes and inducing cyclin D1 transcription in invertebrates^55,56^ and vertebrates^32^. Thereby, cJUN negatively regulates p53 expression by binding to its promoter, thereby promoting cell cycle progression and proliferation^57^. Nematosomes are budding from distinct regions of the mesenteries into the gastric cavity^27^. In addition to the proliferation of nematosomes, the mesenteries of *N. vectensis* play a crucial role in endomesodermal patterning and germ cell development^58,59^, demonstrating the highly proliferative properties of this tissue. Our results show that cJUN is essential for controlling the expansion of nematosomes, potentially by regulating genes involved in cell division and differentiation, suggesting that cJUN not only governs phagocytosis but also orchestrates the development and maintenance of nematosomes as functional immune units. In addition to adult polyps, reduced nematosomes abundance was also observed in egg packages derived from *cJUN*^−/−^ females and was associated with reduced offspring fitness in a mother genotype-dependent manner. These observations are consistent with the hypothesis that maternal provisioning of nematosomes contributes to transgenerational immune protection. However, because *cJUN* is expressed in multiple tissues, we cannot exclude that additional cJUN-dependent maternal effects contribute to the reduced offspring survival. The loss of cJUN function also resulted in microbial dysbiosis, with mutant polyps displaying altered microbiome compositions dominated by non-native bacterial species. These findings establish a direct link between cJUN-mediated immune regulation and microbiome homeostasis in *N. vectensis*. In Drosophila, JNK signaling, which activates c-Jun, is essential for innate immunity and development^60^. In mammals, epidermal JunB regulates cutaneous immune cell-microbiota interactions, with its absence leading to atopic dermatitis-like symptoms and spontaneous *S. aureus* colonization^61^. c-JUN/AP-1 is particularly important in CD8 T-cell responses to acute infection, participating in productive immune responses^62^. These studies highlight the complex interplay between cJUN-mediated signaling, immune regulation, and microbiome homeostasis in various organisms and contexts. Recent findings in sponges further support an evolutionarily conserved role in cJUN/AP-1 in symbiont recognition. In *Amphimedon queenslandica*, symbiotic bacteria induce a rapid transcriptional response involving cJUN/AP-1, NF-κB and IRF, whereas foreign bacteria do not trigger these responses and instead elicit xenobiotic metabolism^63^. This mirrors our observations in *N. vectensis* and suggests that ancient metazoans already employed AP-1 family transcription factors to distinguish beneficial symbionts from potentially harmful bacteria, maintaining holobiont stability through selective immune regulation.

Together, these observations indicate that cJUN-dependent regulation of nematosomes influence host fitness across multiple life stages, from embryonic development to adult microbial homeostasis.

### Innate immune specificity and its implications for host-microbe interactions

The strong evidence for innate immune specificity in *N. vectensis* has significant implications for our understanding of the evolution of host-microbe interactions. Traditionally, innate immunity has been viewed as a broad, non-specific defense mechanism, whereas adaptive immunity is considered the primary driver of immune specificity. However, our findings refine this distinction by showing that selective microbial discrimination can arise through innate cellular mechanisms in early-diverging animals, like cnidarians. The ability of nematosomes to distinguish between closely related bacterial strains and selectively regulate microbiome composition suggests that innate immune specificity is an ancient and fundamental feature of metazoan immunity. Interestingly, we also observed differences among the native *Vibrio* isolates themselves, indicating that nematosome responses are not only able to distinguish between native and foreign bacteria, but also discriminate at the strain level. Similar strain-specific host responses have recently been described in the squid-Vibrio symbiosis^64^, where closely related bacterial strains triggered distinct host transcriptional and developmental programs depending on their evolutionary and ecological origin. These findings support the idea that fine-scale microbial recognition and differential host responses to closely related bacterial strains may represent a conserved feature of host-microbe interaction across diverse symbiotic systems. This aligns with recent studies in other invertebrates, which have also demonstrated a surprising degree of innate immune selectivity, further supporting the notion that immune specificity predates vertebrate adaptive immunity^38,65,66^. In several studies, it was shown that invertebrates can differentiate between pathogens at the species and even strain level^67,68^. This specificity is particularly evident in immune priming, in which initial exposure to a pathogen confers protection against subsequent encounters. Immune priming in invertebrates is a phenomenon in which an initial exposure to a pathogen enhances immune defenses against subsequent infections. This adaptive-like immunity has been observed in various invertebrates^69,70^, such as in woodlice^9^, where hemocytes show increased phagocytosis of previously encountered bacterial strains. In the oyster *Crassostrea gigas*, hemocytes exhibit differential phagocytic responses to various bacterial species, demonstrating that invertebrate immune cells can selectively recognize and respond to different microbes^71,72^. Similarly, in the squid-Vibrio symbiosis, host immune cells differentiate between preferred symbionts and other closely related bacteria^17^. In addition, immune priming can be enhanced by protective symbionts^73^. Potential mechanisms involved are sustained immune responses, epigenetic modifications, and metabolic reprogramming, though the underlying mechanisms are not fully understood^74^. However, C-type lectin-like domain (CTLD) proteins have been identified as potential contributors to this specificity, with their extreme gene diversification observed in various invertebrate genomes^75^. *Drosophila* exhibits a specific primed immune response against certain pathogens based on phagocytosis^76^ that requires phagocytes and the Toll pathway. This priming involves exposure to dead or sublethal doses of microbes, eliciting an initial response that enhances protection against subsequent infections^77^. These examples provide additional support for the concept that innate immune specificity is an ancient and widespread phenomenon across diverse metazoans.

The evolutionary advantage of innate immune specificity likely lies in its ability to balance microbial diversity while preventing colonization by poorly compatible or potentially harmful bacteria. In the case of *N. vectensis*, the selective phagocytosis of foreign bacterial isolates by nematosomes likely contributes to maintaining a stable and beneficial microbiome. At the same time, our pathogen challenge experiments indicate that this system may also become particularly important during acute infection, as cJUN-deficient animals showed increased susceptibility to *V. coralliilyticus*. This suggests that nematosomes may function in both microbiome homeostasis and pathogen defense depending on the microbial context. This mechanism is particularly crucial for organisms with simple immune architectures, where adaptive immune responses are absent. By employing a finely tuned innate immune response, *N. vectensis* can maintain a dynamic yet controlled microbiome, allowing for environmental adaptability^24^ without compromising immune defenses. This study, therefore, positions cnidarians as valuable models for exploring the evolutionary origins of immune-microbe interactions and provides insights into how early metazoans may have developed mechanisms for microbial regulation through selective innate immunity in the absence of adaptive immunity.

In conclusion, our study provides compelling evidence that *N. vectensis* employs a selective innate immune mechanism to regulate its microbiome, refining the traditional perception of innate immunity as solely a non-specific defense. The role of nematosomes in selectively phagocytosing foreign bacteria and the involvement of cJUN in orchestrating this process highlight the molecular complexity of immune regulation in early metazoans. Our findings reinforce the idea that innate immune specificity is evolutionarily ancient and widespread among invertebrates, playing a crucial role in maintaining host-microbe homeostasis and contributing to host defense under pathogenic challenge. By demonstrating the impact of selective immune responses on microbiome composition, our work contributes to a broader understanding of host-microbe interactions and their evolutionary significance. Future research should further explore the molecular pathways underlying nematosome-mediated immunity and examine how these mechanisms have influenced the evolution of immune systems across metazoans.

## Methods

### Nematostella vectensis culture

All experimental setups were conducted with the F1 adult clonal female *N. vectensis* offspring of CH2XCH6, originally collected from the Rhode River in Maryland, United States^78,79^. The polyps were fed daily with freshly hatched *Artemia nauplii* and kept in the dark. Culture tanks containing the animals, separated by genotype and gender, were connected to an aquatic system where the medium was flushed out and replaced with fresh *Nematostella* Medium (NM) with a salinity of 16‰ (Red Sea Salt® and Millipore H2O) at 18 °C every other day. Every two weeks, the culture boxes were manually cleaned to remove biofilm and feeding debris.

### Isolation of nematosomes from adult polyps and egg packages

For quantification of nematosomes abundance *cJUN*^−/−^ and *cJUN*^*+/+*^ polyps, size-matched adult animals were collected and transferred to dishes containing 16‰ NM. A small incision was made in the foot region using fine forceps, allowing nematosomes to be released from gastric cavity. Extruded nematosomes were collected using a pipette, transferred to microscope slides, and imaged for subsequent quantification using ImageJ Fiji^80^.

To quantify the amount of nematosomes in egg packages, adult polyps were induced to spawn by overnight incubation at 25 °C under light exposure for 11 h (Supplementary Fig. 1a). Egg packages were collected, and the surrounding gelatinous matrix was removed by incubation in 4% cysteine solution in 16‰ NM for 20 min at room temperature. Released eggs were imaged and counted. The remaining suspension containing eggs and nematosomes was subsequently passed through a 70 µm cell strainer (pluriSelect) to separate eggs from nematosomes. The flow-through containing nematosomes was collected, and nematosomes were counted microscopically.

### Phagocytosis assay and dual-strain bacterial challenge

Experimental setups for bacterial challenges were performed using the same bacterial isolates used for mono-associations. Native (NJ1, NJ33 and NA11)^26^ and non-native (Hal025 and Hal281^38^) (Supplementary Table 1) bacterial isolates were grown at 30 °C and 200 x g overnight in liquid Marine Broth (MB) Medium before diluting the isolates to an OD600 of 0.1. 1 mL of bacteria was centrifuged and diluted with sterile 16‰ NM prior to staining with BacLight (0.1 µM, Thermo Fisher) for 15 min in the dark and at room temperature. After incubation, bacteria were centrifuged for one wash step with 16‰ NM before diluting them to OD600 of 0.001 for the bacterial challenge on the nematosomes ex vivo. Bacterial treatment was performed in the dark for 1.5 h at 18 °C.

During bacterial staining, nematosomes from five clonal female polyps were extracted by pinching a hole in the food region of the polyps and pipetting the discharged nematosomes, and placed into a chamber slide (Thermo Scientific™ Nunc™ Lab-Tek™ II Chamber Slide™ System). After letting the cells stick to the bottom of the slide, they were washed once with NM to get rid of debris. The nematosomes were treated with stained bacteria from an OD600 of 0.001 and incubated for 2 h in the dark. After bacterial treatment, nematosomes were washed two times with 16‰ NM. After that, nematosomes were stained with LysoTracker (15 nM, Thermo Fisher) and Hoechst (10 nM, Thermo Fisher) for 45 min at room temperature. Staining solution was washed out with NM after staining. Cells were than fixed with 3 % PFA diluted in 16‰ NM for 15 min on RT and washed out once with NM after treatment. Nematosomes were mounted on a slide using ProLong™ Diamond (Thermo Fisher). Images were taken with the Olympus FV3000 Confocal Laser Scanning Microscope. For the quantification of lysosomal positive area, the LysoTracker-positive area was measured and normalized to the total nematosome area using image analysis in ImageJ Fiji. The resulting values are presented as the percentage of LysoTracker-positive area relative to the total nematosome area (%).

For simultaneous bacterial challenge experiments on extracted nematosomes, one bacterial isolate from the native group (NJ1, NJ33, or NA11) and one isolate from the foreign group (Hal025 or Hal281) were fluorescently labeled with different BacLight dyes. Native isolates were stained with BacLight Green (0.1 µM), whereas foreign isolates were stained with BacLight Red (0.1 µM, Thermo Fisher). Bacterial cultures were grown as described above and adjusted individually to an OD600 of 0.001 prior to staining. Fluorescent labeling was performed for 15 min in the dark at RT, followed by one wash with 16 ‰ NM. Nematosomes were extracted and transferred into a chamber slide as described previously. Equal volumes of the native and foreign bacterial suspension were mixed (1:1 ratio) and added to the nematosomes. Cells were incubated with both bacterial strains simultaneously for 1.5 h at 18 °C in the dark. After incubation, nematosomes were washed once with NM to remove unengulfed bacteria. Nematosomes were then fixed with 3% PFA diluted in NM for 15 min at RT, washed once with NM, and mounted using ProLong Diamond. Images were acquired using confocal laser scanning microscope.

### Generation of germfree polyps, mono-associations and pathogen infection

Antibiotic treatment (AB treatment) approaches were adapted from the established protocol for generating germfree *Hydra* polyps^81^ and further adapted for *Nematostella*^82^ (Fig. 2d, Supplementary Fig. 6a). Adult clonal *cJUN*^*+/+*^ and *cJUN*^−/−^ lines were exposed to a combination of five antibiotics: Ampicillin, Neomycin, Streptomycin, Spectinomycin, and Rifampicin, each at a concentration of 50 µg/mL. This treatment was conducted over a period of 2 weeks without any food supply, with the medium being refreshed every day and a replacement of plates every second day. For each treatment condition, five biological replicates were utilized, along with an additional five biological replicates serving as germfree (GF) and wildtype (WT) controls. Following the 2-week AB treatment, polyps were washed in sterile, filtered 16 ‰ NM before homogenization. A 1:10 dilution of the lysate was plated on Marine Broth (MB) agar plates to confirm sterility, in which GF plates should remain clear without bacterial growth. The remaining lysate was centrifuged, and the pellet was processed for DNA isolation using the Qiagen Blood and Tissue Kit for subsequent molecular analyses, including PCR and quantitative PCR (qPCR). After confirming sterility, the remaining polyps were prepared for mono-association with the chosen bacterial isolates. After 2 weeks of AB Treatment, polyps remained in sterile and filtered NM prior to recolonization. Bacterial isolates were grown at 30 °C in liquid MB media overnight. Bacteria were grown to an OD600 of 0.1, diluted to a final OD600 of 0.001 and exposed to the sterile polyps. We used four polyps per isolate and genotype, with GF and WT controls, respectively. The isolates we chose were NJ1, NJ33 and NA11 as native colonizers for *Nematostella* and Hal025 and Hal281 as non-native, foreign, colonizers, obtained from *Halichondria panicea*. For pathogen challenge experiments, polyps were exposed to the pathogen *V. coralliilyticus*^37^ (Supplementary Table 1). Following mono-association with individual bacterial isolates, sampling took place after 2- and 7- days post recolonization (2 dpr and 7 dpr). To quantify bacterial colonization, a subset of polyps was washed three times with 16 ‰ NM before being homogenized and plated on MB plates. After an incubation time of 2 days and 7 days on RT, colony-forming units (CFU) were counted manually to assess bacterial colonization levels. A second subset of recolonized and pathogen-treated polyps was monitored to assess survival performance, and animals were tracked until death. In a separate experiment, polyps were observed longitudinally to assess health status using a defined health-scoring system (Supplementary Fig. 3).

### Health scoring of bacterial-challenged polyps

Polyp health was assessed using a morphology-based scoring system based on a predefined reference catalog representing progressive stages of deterioration (Supplementary Fig. 3). The catalog comprised six scoring categories, ranging from one (healthy animals) to 6six (severely deteriorated animals). At each observation time point, individual polyps were transferred from the culture room to the microscope, allowed to relax sufficiently, and visually assessed based on four independent morphological parameters: body length, body fluid, mesentery condition, and tentacle morphology. Each parameter was assigned a score from 1 to 6 according to the reference catalog, where lower scores indicate healthy, extended animals and higher scores indicate increasing levels of morphological decline. To obtain a single quantitative health metric per animal, the scores of four parameters were averaged to calculate a mean health score for each polyp. This approach integrates multiple morphological indicators of an animal’s condition into one composite value. Six biological replicates were analyzed per treatment group and genotype, and polyps were scored longitudinally throughout the experiment to monitor changes in health following bacterial recolonization and pathogen challenge.

### Proteomic analysis

Bacterial culture was prepared with an OD600 of 0.001 (see Phagocytosis assay) prior to nematosomes extraction from adult polyps. Nematosomes from five biological replicates per treatment were collected. The bacterial isolate NJ1 was used as a native colonizer of *N. vectensis*, whereas Hal281 was used as a non-native isolate. In addition, a control group without bacterial challenge was included. Extracted nematosomes were treated for 2 h at 18 °C with the isolates and were washed afterward with 16 ‰ NM once. Nematosomes were transferred into a PCR tube and centrifuged at 2800 x g for 5 min at 4 °C to collect in the bottom of the tube. NM was discarded and replaced with 25 µl Lysis Buffer (5 mol/L Urea, 1 % Tritonx100, 1 x cOmpete EDTA-free, 5 mmol/L DTT). Nematosomes were incubated for 45 min at 37 °C with mixing every 15 min. After incubation, nematosomes were frozen at -80 °C for further analysis. Samples were then digested according to SP3 protocol^83^ with some modifications as follows. After thawing, lysates were mixed each with 25 µL Alkylation buffer (50 mM borate buffer and 25 mM IAA) for 50 min at room temperature (RT). Then, 5 µL of resuspended SP3 beads (20 µg/µL A:B 1:1 mixture) were added to each sample followed by 150 µL ACN and mixed for 30 min at 100 x g, RT. Then, beads were washed with 300 µL 70 % EtOH, followed by 150 µL ACN. A 10 µL digestion buffer (4 ng/µL trypsin/Lys-C, 25 mM borate buffer and 0.01 % DDOPM) was added to each sample, followed by mixing on a shaker for 10 min at 100 x g RT. Samples were mixed by pipetting up and down and kept on the shaker overnight. The next day, samples were centrifuged at 20,000 x g for 2 min and supernatant (ca. 10 µL) was transferred to LC-MS vials containing 1 µL of 5 % FA.

### LC-MS proteomics and data analysis

Chromatographic separation was performed on a Dionex U3000 nanoHPLC system equipped with an Acclaim pepmap100 C18 column (2 μm particle size, 75 μm × 500 mm) coupled online to a mass spectrometer. The eluents used were: eluent A: 0.05% formic acid (FA), eluent B: 80 % ACN + 0.04 % FA. The separation was performed over a programmed 120 min run. Initial chromatographic conditions were 4 % B for 2 min followed by linear gradients from 4 to 50% A over 90 min then 50– 90% A over 5 min, and 10 min at 90% A. Following this, an inter-run equilibration of the column was achieved by 16 min at 4% A. A constant flow rate of 300 nl/min was employed. Data acquisition following separation was performed on an QExactive Plus. Full scan MS acquisition was performed (350–1400 m/z, resolution 70,000). Subsequent data-dependent MS/MS scans were collected for the 15 most intense ions (Top15) via HCD activation at NCE 27.5 (resolution 17,500); dynamic exclusion was enabled (20 sec duration). Triplicate measurements were performed for all the samples.

Raw data were analyzed against *Nematostella vectensis* Uniprot database (20.05.2022) (24,497 sequences) plus common contaminants (cRAP). The search was performed on Proteome Discoverer 2.5 using a SequestHT search engine with 10 ppm and 0.02 Da precursor and fragment ions tolerances, respectively. Digestion with trypsin, with a max of two missed cleavages, was applied. Strict parsimony criteria have been applied, filtering peptides and proteins at 1% FDR. INFERY's rescoring algorithm was applied. A label-free quantification method based on precursor ion intensities was used. Proteins were filtered to have “High” FDR combined confidence and at least two identified peptides. Data was further analyzed by Excel and Perseus v 1.6.15.0^84^. Protein intensities were averaged for technical replicates. To perform differential quantitative analysis of proteins, raw protein intensities were extracted, averaged between technical replicates, one outlier replicate per group “Control 5, Native 1 and Non-Native 2” were excluded, then median based normalization was applied to the data. Log2-transformed intensities were grouped in 3 groups depending on the *Vibrio* treatment (each with 5 replicates). Proteins with at least 4 intensity values in one group were used for further analysis. Missing values were imputed from a normal distribution separately for each replicate (Width 0.3, Downshift 1.8). Statistical analysis was done using ANOVA, permutation-based FDR of 0.01. Gene enrichment analysis was performed on the Database for Annotation, Visualization, and Integrated Discovery (DAVID)^85,86^ using the functional annotation tool.

### CRISPR/Cas 9-mediated knock-out generation

CRISPR/Cas9-mediated transgenic lines were generated following the protocol published before^40^. The gene *cJUN* (NVE21090) was selected based on transcriptomic analysis after recolonization experiments^26^ and tissue specificity^27^. Four guide sequences (sgNVE21090E1, sgNVE21090E2, sgNVE21090E3, and sgNVE21090E4) were designed using the web tool CRISPOR.org^87^, with an implemented *Nematostella* genome. First, the guide oligonucleotides were mixed in equal amounts, annealed for 5 min at 95 °C, and then incubated at room temperature for 2–3 h. The annealed oligos were cloned into the gRNA expression vector pDR274 (42250, Addgene). After successful integration of the guides into the vector, the guide sequences were amplified, transcribed in vitro using the MEGAscript™ T7 kit (Thermo Fisher), and purified with the MEGAclear RNA cleanup kit (Thermo Fisher) prior to injection. The injection mix consisted of Cas9 enzyme (1 mg/ml stock) (TrueCut™ Cas9 Protein v2, Thermo Fisher), sgRNAs (450 ng), Alexa fluorescent dye (1.1 M in KCl), and RNase-free water. To obtain fertilized eggs, animals were incubated at 25 °C for 11 h to induce gamete production. Egg packages were incubated in sperm media from males for 15 min before dejellying the fertilized egg packages with 4% cysteine (pH 7.4), followed by five washing steps in 16‰ NM. The injection mix was incubated at 3 °C for 5 min before injection. The injection setup was conducted according to the microinjection protocol for mRNA and Morpholinos previously described^88^. Injected eggs were raised in the dark at 20 °C, with the medium (NM) being exchanged daily, and food introduction after 10–12 days.

To confirm the successful integration of CRISPR/Cas9-mediated mutagenesis into the polyps’ genome, we performed crossbreeding, High Resolution Melting Curve Analysis (HRMC) and genotyping. Genomic DNA was isolated from injected juvenile polyps using the DNeasy Blood & Tissue kit (Qiagen). In HRMC, short DNA fragments at the targeted locus are amplified, and changes in these fragments are detected through shifts in the melting curves. Following confirmation of successful mutations, the mutant animals were crossed with wild-type polyps to generate the F1 generation. The same procedure was applied as for the previous F0 generation. Heterozygous animals were further analyzed by Sanger sequencing to determine the precise mutation pattern. Polyps with the same mutation pattern were crossed to generate the F2 generation, which included homozygous mutants (*cJUN*^−/−^), homozygous wild-type (*cJUN+/+*), and heterozygous (*cJUN+/−*) offspring, which are used in experimental setups.

### Electron microscopy with nematosomes

Scanning electron microscopy (SEM) was taken with the Zeiss REM Supra 55VP. Nematosomes were extracted from the polyps and placed on a well with Poly-L-Lysine-coated cover glasses. All fixation, washing, dehydration were performed as former published^89^.

Samples were first fixed for 1 h at RT in 4 % glutaraldehyde prepared in 0.1 M Na cacodylate buffer, followed by three washes of 10 min each in 0.1 M cacodylate buffer. A second fixation step was then carried out for 1 h in the dark at 25 °C using 2% osmium tetraxide (in 0.2 M cacodylate buffer). After fixation, samples were washed three times for 10 min each in 0.1 M cacodylate buffer. Dehydration was performed through a graded ethanol series beginning with 30% ethanol for 10 min, followed by 50% ethanol for 2 x 15 min, 70% ethanol for 15 min, and an additional 70% ethanol overnight, all at RT. On the following day, dehydration was continued with 80% ethanol for 2 x 15 min, 90% ethanol for 2 x 15 min, 96% ethanol for 2 x 15 min, and finally 100% ethanol for 2 x 15 min, all at RT. Samples were subsequently critical point dried, mounted, and sputter coated prior to imaging. Acquired images were analyzed using ImageJ Fiji.

### 16S rRNA analysis

For 16S rRNA analysis, the bioinformatics were performed using Qiime 2 2021.11^90^. First, raw sequences were demultiplexed and quality filtered using the q2-demux plugin, followed by denoising with DADA2^91^. The amplicon sequence variants (ASVs) were aligned with mafft and fasttree2 was used to construct the phylogeny^92,93^. All samples were rarefied to 900 sequences per sample prior to estimation for Alpha-diversity metrics (observed features and Faith’s Phylogenetic diversity^94^), beta-diversity metrics (Bray-Curtis dissimilarity, Jaccard distance and Unifrac (weighted and unweighted^95,96^)) and Principal Coordinate Analysis (PCoA). Taxonomy was assigned to ASVs using q2-feature-classifier classify-sklearn naïve Bayes taxonomy classifier against Greengenes 13_8 99% data set as reference^97^. Further analysis, statistical analysis and plot visualization were conducted with OriginPro (Version 2021. OriginLab Corporation, Northampton, MA, USA.).

### Statistics & reproducibility

Statistical analyses were performed using OriginPro 2024 and QIIME2 (v. 2021.11), as appropriate for each analysis. Image quantification was performed using Fiji/ImageJ. Unless otherwise stated, all statistical tests were two-sided. No statistical method was used to predetermine sample size. Sample sizes were based on previous experience with *Nematostella vectensis* microbiome experiments. Unless otherwise stated, all reported sample sizes (*N*) refer to biologically independent experimental units (e.g., polyps, nematosomes or bacterial cultures, as appropriate) and are indicated in the corresponding figure legends. No data were excluded from the analyses unless a technical failure occurred during sample preparation, imaging, or sequencing. Animals from the same culture conditions were randomly distributed across experimental groups where possible. Investigators were not blinded to allocation during experiments or outcome assessment because the experimental treatments were apparent during sample preparation and imaging. Where representative microscopy images are presented, they were selected from independent biological preparations that yielded similar results.

## Supplementary information

Supplementary Information

Description of Additional Supplementary Files

Dataset 1

Dataset 2

Dataset 3

Supplementary Movie

Transparent Peer Review file

## Source data

Source Data

## Data Availability

Source data and Supplementary information files are provided with this paper. Raw 16S rRNA gene amplicon sequencing data generated in this study have been deposited in the NCBI Sequence Read Archive under the BioProject number PRJNA1464507. The mass spectrometry proteomics data have been deposited to the ProteomeXchange Consortium (http://proteomecentral.proteomexchange.org) via the PRIDE partner repository^98^ with the dataset identifier PXD065753. No restrictions apply to data access. Source data are provided with this paper.
