# Supplementary material for: c-JUN controls microbial colonization via selective phagocytosis in the sea anemone Nematostella
Source: Nat Commun. 2026 Jul 10;17:6087. doi: 10.1038/s41467-026-75511-w (PMC13354778; doi:10.1038/s41467-026-75511-w)
Supplement: Supplementary file 1 — Supplementary Information [file 41467_2026_75511_MOESM1_ESM.pdf]

Supplementary Information

c-JUN controls microbial colonization via selective phagocytosis in the sea anemone Nematostella

Kaya N. H.[1], Abukhalaf M.[2], Fuentes G.[1], Taubenheim J.[1,3], Hentschel U.[4], Tholey A.[2], Fraune S.[1*]

Table S1. **Bacterial isolates used in this study and their taxonomic classification.** Overview of all bacterial isolates used for recolonization, phagocytosis, and pathogen challenge experiments in *Nematostella vectensis*. The table includes isolate origin (host organism), experimental role (native, foreign, or pathogen), available GenBank accession numbers, closest related type strains based on 16S rRNA gene sequence similarity, percentage sequence identity, corresponding ASV assignments according to Domin et al. 2023, and original references describing the isolates. Native isolates (NJ1, NJ33, and NA11) were originally isolated from *N. vectensis*, whereas foreign isolates (Hal025 and Hal281) originated from the sponge *Halichondria panicea*. *Vibrio coralliilyticus* was used as a pathogenic challenge strain in infection experiments obtained from *Pocillopora damicornis*.

**Isolate**	**Host**	**role in this study**	**GenBank accession number (if available)**	**Closest type strains (with Sequence ID)**	**Similarity (%)**	**ASV number according Domin et al 2023**	**Reference**
NJ1	*Nematostella vectensis*	*native*	PQ455196	*Vibrio sp. BJGMM-B31* (JQ716236.1)	99,72%	23	Domin et al 2023
NJ33	*Nematostella vectensis*	*native*		*Vibrio diazotrophicus* (CP151842.1)	99,44%	59	Domin et al 2023
NA11	*Nematostella vectensis*	*native*		*Vibrio plantisponsor* (AP024893.1)	98,74%	19	Domin et al 2023
Hal025	*Halichondria panicea*	*foreign*		*Vibrio gigantis* (AP025493.1)	99,86%		
Hal281	*Halichondria panicea*	*foreign*	MT406665	*Vibrio cyclitrophicus* (MT406665.1)	99,87%		Marulanda-Gomez et al 2025
Vibrio coralliilyticus	*Pocillopora damicornis*	*pathogen*	AJ440005	*Vibrio coralliilyticus* (NR028014.1)	100%		Y. Ben-Haim et al 2002

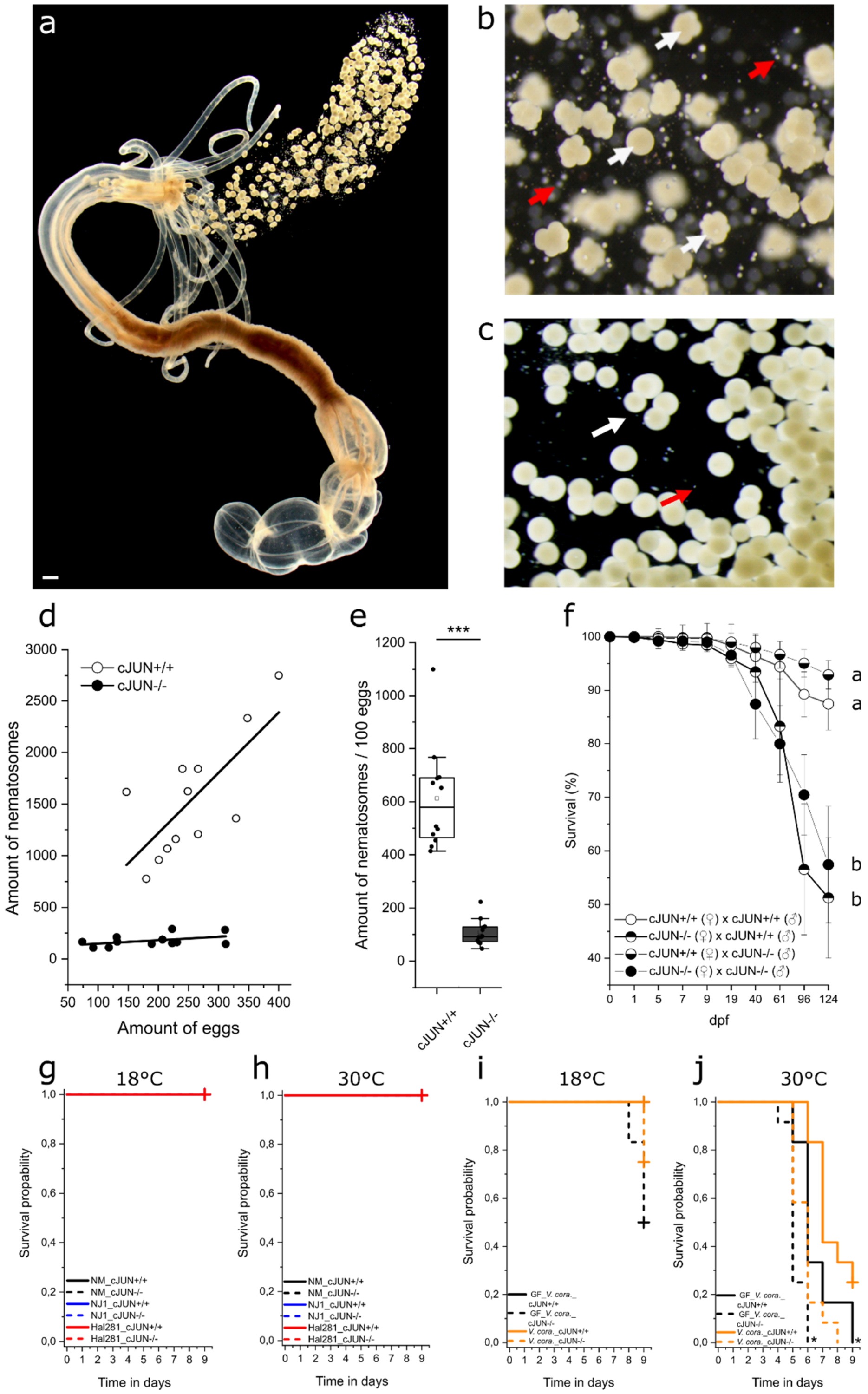

Figure S1. **Maternal nematosomes contribute to early-life immune defense in *N. vectensis*.** **a** Adult female *N. vectensis* releasing egg package. Scalebar 2 mm. **b** Egg package from *cJUN*$^{+/+}$ animal with eggs representing different cleavage status (white arrows) and nematosomes (red arrows) in between the eggs covered in the matrix. **c** Egg package from *cJUN*$^{-/-}$ animal with eggs

(white arrow) and nematosomes (red arrows). Representative images from 20 independent biological preparations with similar results is shown (**a, b, c**). **d** Linear regression analysis of nematosomes abundance as a function of egg number in *cJUN*$^{+/+}$ and *cJUN*$^{-/-}$. Each point represents an individual egg package. Open circles indicate *cJUN*$^{+/+}$ eggs, and filled circles represent eggs released from *cJUN*$^{-/-}$ female mutants. The solid lines indicate linear regression fits. In *cJUN*$^{+/+}$, nematosome abundance increased significantly with egg number (linear regression, $F_{(1,10)} = 11.48$, $R^2 = 0.53$, $p = 0.0069$). In contrast, no significant relationship between egg number and nematosome abundance was observed in *cJUN*$^{-/-}$ animals ($F_{(1,10)} = 2.54$. $R^2 = 0.20$, $p = 0.1418$). N = 12 egg packages / genotype**.** **e** The number of nematosomes per 100 eggs was significantly higher from *cJUN*$^{+/+}$ egg packages compared to *cJUN*$^{-/-}$ mutants. N = 12 egg packages / genotype, Mann-Whitney U test ($U = 144$, $Z = 4.13$, $p < 0.0001$***). Box plots indicate median (center line), interquartile range (box), and range (whiskers). **f** Long-term survival of offspring derived from *cJUN*$^{+/+}$ and *cJUN*$^{-/-}$ mothers. Survival was monitored over 124 days (days post fertilization (dpf)). Data represent mean ± SEM. Statistical analysis using two-way ANOVA revealed significant effects of maternal genotype ($F_{(1,240)} = 80.41$, $p < 0.0001$), time ($F_{(9,240)} = 165.70$, $p < 0.0001$) and their interaction ($F_{(27,240)} = 19.70$, $p < 0.0001$), indicating diverging survival trajectories between groups over time. Different letters indicate statistically significant differences between groups based on Bonferroni multiple comparison ($p <= 0.05$). N = 7 egg packages / group. **g** and **h** survival of animals exposed to native (NJ1) and foreign (Hal281) bacterial isolates compared to non-bacterial challenge (NM) at 18 °C **(g)** and 30 °C **(h)**. No mortality was observed within the observation period under these conditions. N = 12 / genotype. **i** and **j** Survival of animals challenged with the pathogen *Vibrio coralliilyticus* (*V. cora.*). Animals were either germfree (GF) or maintained under conventional conditions and monitored at 18 °C **(i)** and 30 °C **(j)**. At 18 °C, survival remained largely unaffected. Statistical differences were assessed with a log-rank test ($\chi^2 (3) = 14.21$, $p = 0.0027$) In contrast, infection at 30 °C caused rapid mortality, with differences in survival dynamics between genotypes and microbiological conditions (log-rank test ($\chi^2 (3) = 27.18$, $p < 0.0001$)). Crosses indicate censored observations. Asterisks denote significant differences between survival curves determined by log-rank tests, * $p <= 0.05$, ** $p <= 0.01$, *** $p <= 0.001$. N = 12 / genotype.

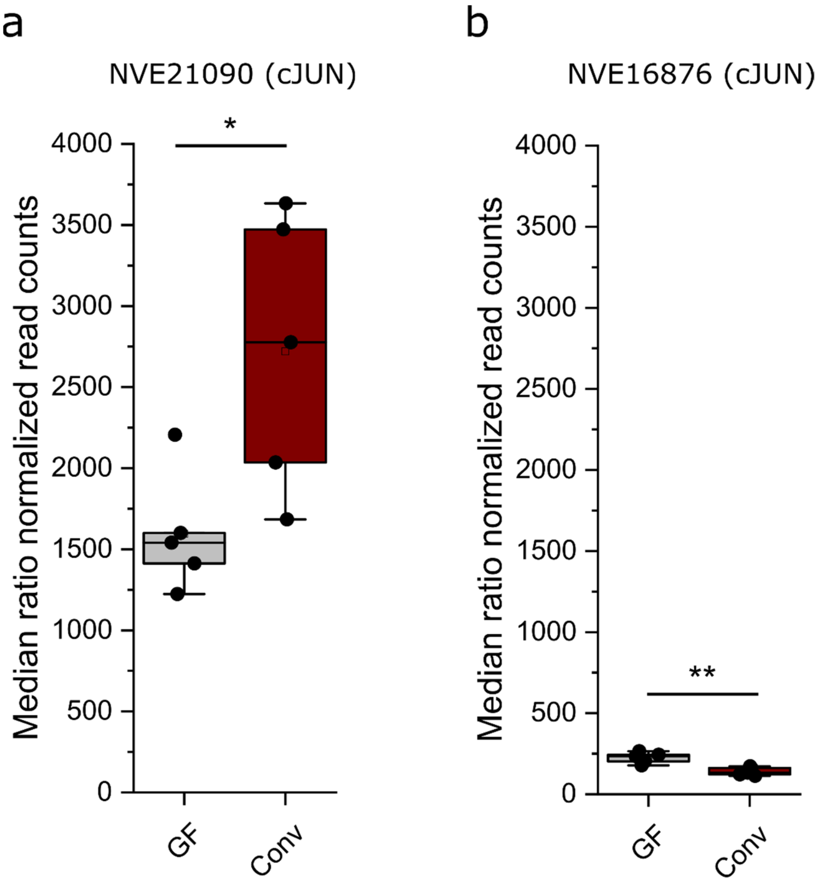

Figure S2. **Bacterial recolonization leads do differentially expression of cJUN orthologs. a** Normalized read counts of NVE21090 in germfree (GF) and conventionalized (Conv) animals. NVE21090 was significantly increased after conventionalization compared to GF controls (two-sided unpaired Students t-test: t = -2.69, df = 8, p = 0.028*. N = 5 polyps. Box plots indicate median (center line), interquartile range (box), and range (whiskers). Points represent individual biological replicates. **b** Normalized read counts of NVE16876 in GF and Conv animals showed reduction in abundance in Conv polyps (two-sided unpaired Students t-test: t = 4.40, df = 8, p = 0.0023**. N = 5 polyps). Box plots indicate median (center line), interquartile range (box), and range (whiskers). Points represent individual biological replicates.

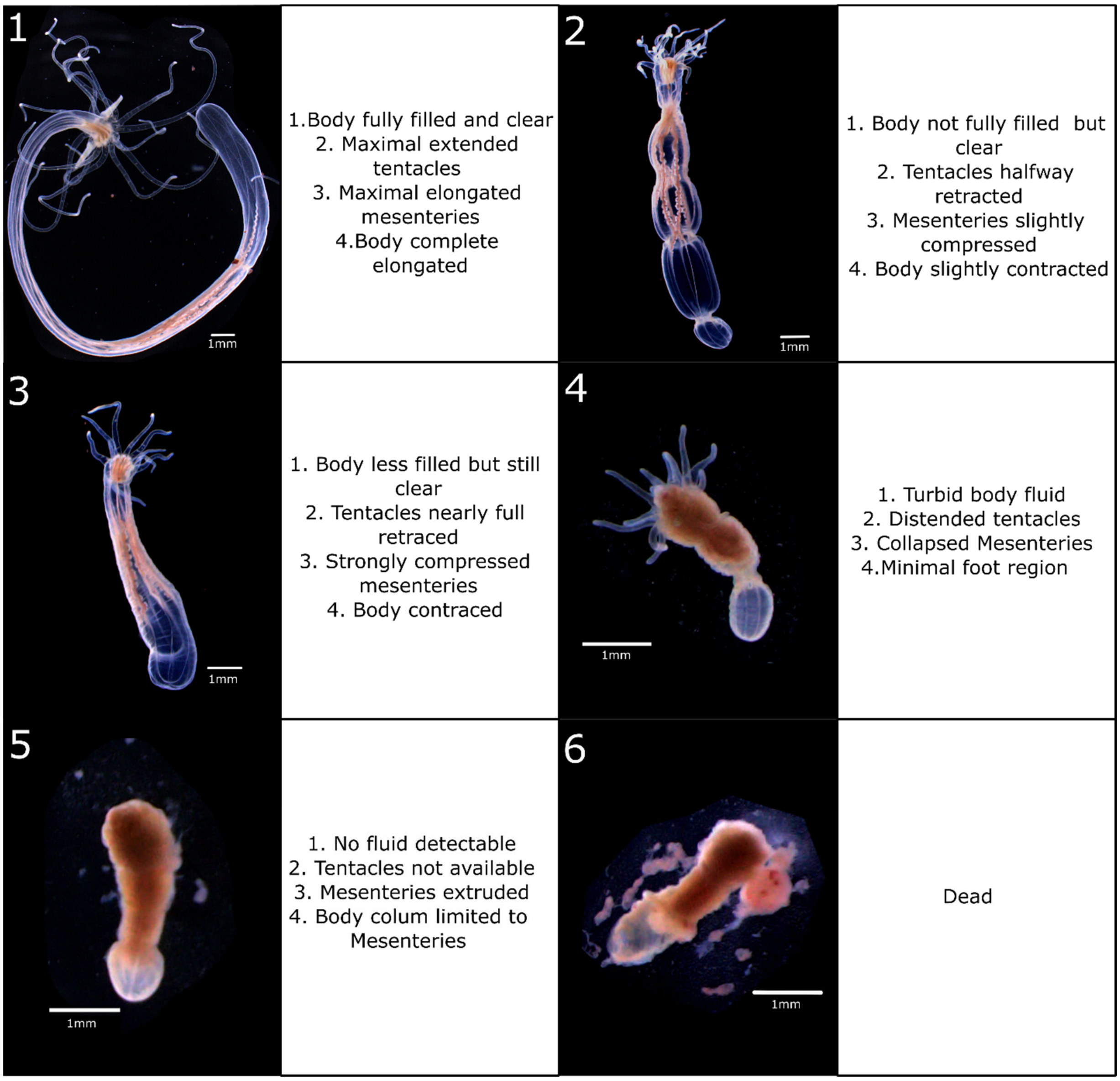

Figure S3. **Health scoring catalogue for *N. vectensis* polyps used to assess animal fitness during experimental treatments.** Representative images illustrating the six health sates used to quantify the physiological condition of *N. vectensis* polyps during the course of the experiments. Animals were evaluated visually under the stereomicroscope and assigned a score ranging from 1 (best condition) to 6 (worst condition) based on morphological criteria reflecting overall tissue integrity and physiological stress. The scoring system was based on four independent characteristics: (i) body length and posture, (ii) body fluidity and tissue transparency, (iii) mesenteries appearance, and (iv) tentacle morphology. Each of these four parameters was scored individually on the same scale from 1-6. The final health score for each polyp was calculated as the mean of these four individual scores, providing an integrated measure of the overall physiological state of the animal. For each treatment condition, six biological replicates were

evaluated. Representative examples of the scoring categories are shown: Score 1 (optimal condition): Polyps display full elongated body column with normal posture and high tissue transparency. Tentacles are extended, flexible, and symmetrically arranged around the oral disc. Mesenteries appear elongated and clearly organized inside the body cavity. Score 2 (very good condition): Polyps remain largely extended but show slight contraction of the body column. Tentacles are still extended but may appear slightly irregular or partially shortened. Mesenteries remain clearly visible. Score 3 (moderate contraction): The body column is shortened and partially contracted. Tentacles are noticeably reduced in extension and may appear curled. Mesenteries remain visible but appear slightly compressed. Score 4 (Strong contraction): Polyps show pronounced contraction with shortened body column and reduced tissue transparency. Tentacles are strongly contracted or partially retraced. Mesenteries appear compressed and less distinct. Score 5 (severe stress): Polyps exhibit strong tissue contraction and compact body column. Tentacles are largely retraced or barely visible, and internal structures such as mesenteries are difficult to distinguish. Score 6 (dead): Polyps display extreme contraction and severe tissue deterioration. Tentacles are absent, mesenteries are no longer discernible, and tissue integrity is strongly compromised. Scalebars represent 1 mm.

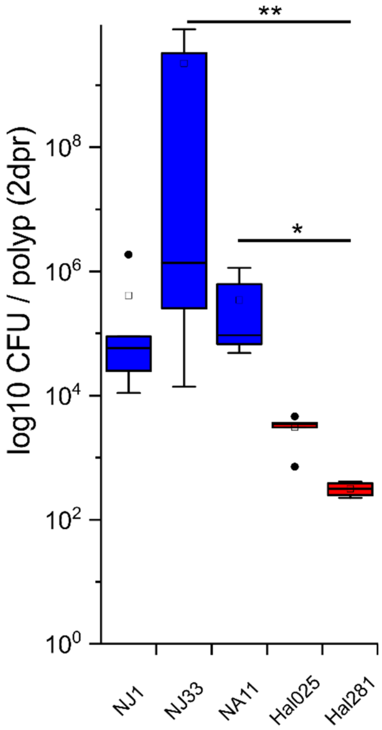

Figure S4**. Differential colonization efficiency of bacterial isolates in adult *Nematostella* polyps at 2 days post recolonization (2 dpr).** Boxplot showing bacterial colonization levels expressed as log10 colony-forming units (CFU) per polyp after recolonization with isolates NJ1, NJ33, NA11 (blue), and Hal025, Hal281 (red). Differences among the isolates were assessed using a Kruskal-Wallis test (χ^2 (4) = 17.72, p = 0.0014) followed by Dunn's multiple comparison. N = 4 (NA11, Hal281), N = 5 (NJ11, NJ33, Hal025) polyps / treatment, * p<=0.05, ** p<=0.01, *** p<=0.001. Box plots indicate median (middle line), interquartile range (box), and range (whiskers), as well as outliers (single points).

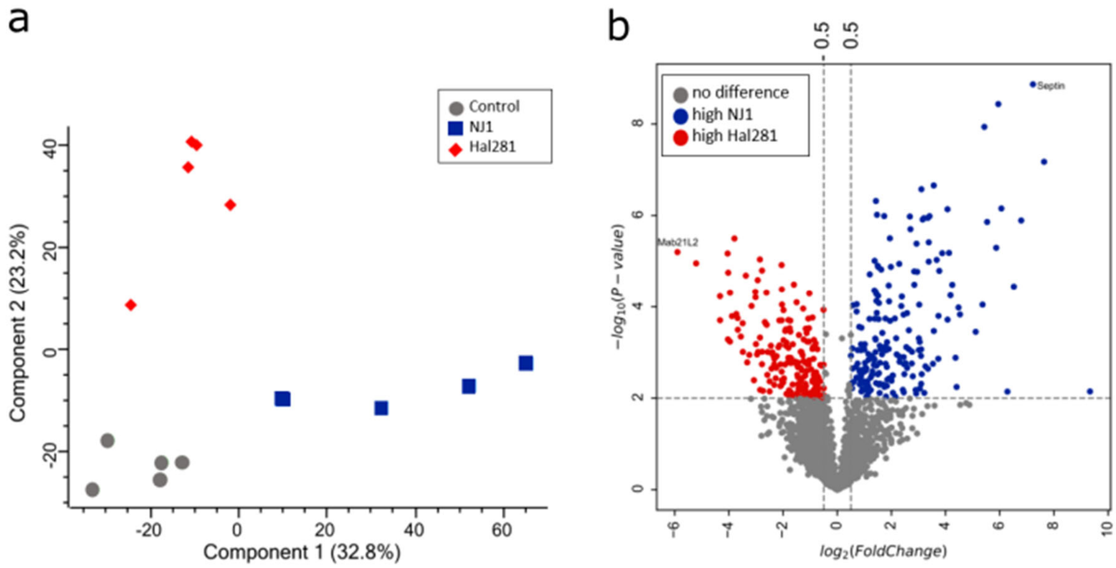

Figure S5. **Proteomic response of nematosomes following treatment with bacterial isolates NJ1 and Hal281. a** Principal component analysis (PCA) of proteomic profiles showing distinct clustering patterns among nematosomes treated with isolates NJ1 (blue), Hal281 (red), and control nematosomes without bacterial challenge (grey). Percentages indicate the explained variance for each principal component. **b** Volcano plot illustrating differentially abundant proteins identified between NJ1- and Hal281-treated nematosomes. Proteins significantly enriched ($p < 0.05$, log_2 fold-change > 0.5) in NJ1-treated nematosomes are shown in blue, those enriched in Hal281-treated nematosomes are in red, and proteins with no significant difference are represented in grey. Selected proteins with strong differential expression are labeled explicitly on the plot.

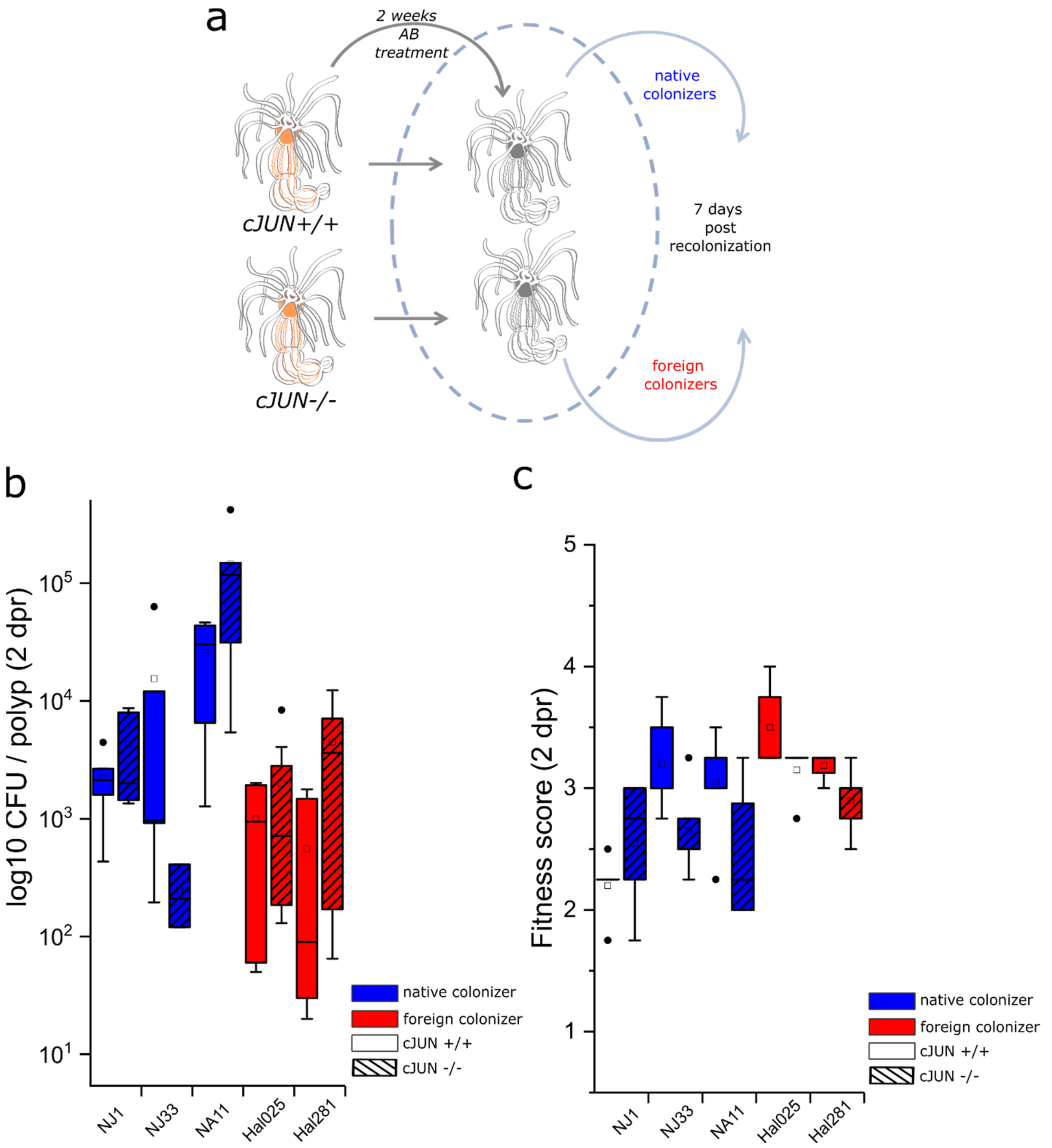

Figure S6**. Prior bacterial exposure modulates nematosomes response to native and foreign colonizers. a** Simplified illustration of AB treatment before mono-association with native and foreign *Vibrio* strains. **b** Mono-association of native and foreign colonizers on adult polyps after 2 dpr. Bacterial colonization levels differed significantly among treatments (Kruskal-Wallis, χ^2 (9) = 26.45, p = 0.0017) followed by Dunn's multiple comparison test. N ($cJUN^{+/+}$) = 6 (NJ1, Hal025), 5 (NJ33, NA11), 7 (Hal281) polyps. N ($cJUN^{-/-}$) = 5 (NJ1, NA11), 3 (NJ33), 8 (Hal025), 6 (Hal281) polyps. Box plots indicate median (middle line), interquartile range (box), and range (whiskers), as well as outliers (single points). **c** Polyp conditions scores at 2 dpr mono-association. Differences

were analyzed using two-way ANOVA with genotype and treatment as factors. Significant effects were detected for genotype ($F_{(1,38)} = 6.60$, $p = 0.014$) and treatment ($F_{(4, 38)} = 8.16$, $p < 0.0001$) while the interaction was not significant ($F_{(4, 38)} = 2.36$, $p = 0.071$). N=5 polyps / genotype. Box plots indicate median (middle line), interquartile range (box), and range (whiskers), as well as outliers (single points).

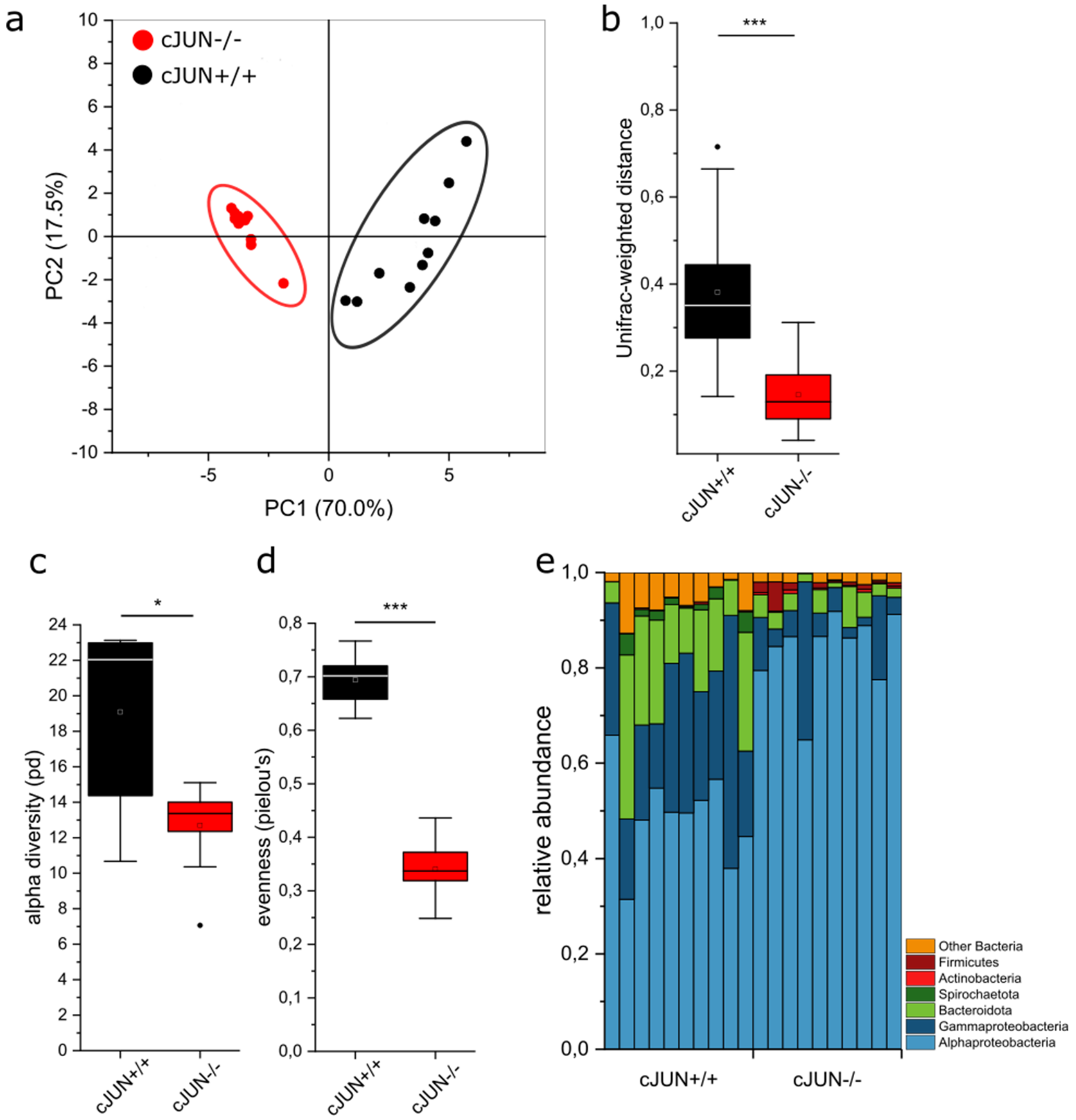

Figure S7. **Impact of *cJUN* KO on microbial diversity and abundance of specific taxa in *Nematostella* polyps. a** Principal Coordinate Analysis (PCoA) plot based on weighted UniFrac distances, comparing the microbial communities of *cJUN*$^{+/+}$ and *cJUN*$^{-/-}$ polyps. The two groups form distinct clusters, indicating significant differences in microbial community composition. N = 10 polyps / genotype. **b** Weighted UniFrac distance demonstrates distinct microbial community composition in *cJUN*$^{-/-}$ versus *cJUN*$^{+/+}$ polyps. Differences in microbiome composition between the genotypes were assessed using a paired Student's t-test (t = 9.66, df = 44, p < 0.0001***). Box plots indicate median (middle line), interquartile range (box), and range (whiskers), as well as

outliers (single points). **c** Alpha diversity analysis using Faith's Phylogenetic Diversity (PD) index revealed a significant reduction in microbiome richness in *cJUN*$^{-/-}$ polyps compared to *cJUN*$^{+/+}$ polyps. N = 10 polyps / genotype, paired t-test (t = 3.20, df = 19, p = 0.0109*). Box plots indicate median (middle line), interquartile range (box), and range (whiskers), as well as outliers (single points). **d** Microbial community evenness (Pielou's evenness index) significantly decreases in cJUN mutant polyps compared to wildtype control. Differences in community evenness between genotypes were assessed using paired t-test (t = 7.12, df = 19, p < 0.0001***). N = 10 polyps / genotype. Box plots indicate median (middle line), interquartile range (box), and range (whiskers), as well as outliers (single points). **e** Bar plots representing the relative abundance of bacterial taxa in *cJUN*$^{+/+}$ and *cJUN*$^{-/-}$ animals. Each bar shows the mean relative abundance of taxa, including Alphaproteobacteria, Gammaproteobacteria, Bacteroidota, Spirochaetota, Actinobacteria, and Firmicutes, across samples for each genotype, highlighting differences in the microbial community composition between the two groups. N = 10 polyps / genotype.

Table S2. **Statistical summary of ADONIS and ANOSIM tests on Bray-Curtis, Jaccard, Weighted UniFrac, and Unweighted UniFrac distance matrices, comparing microbial community dissimilarities between *cJUN*$^{+/+}$ and *cJUN*$^{-/-}$ animals.** The analysis was performed at the genotype level. Adonis R^2 values represent the proportion of variance explained by genotype while ANOSIM R values indicate the degree of separation between the groups, 999 permutations. Significant differences are indicated by p-values, with higher R values reflecting stronger microbial dissimilarities between *cJUN*$^{+/+}$ and *cJUN*$^{-/-}$ polyps.

Parameter	Metric	Adonis R²	Adonis p	ANOSIM R	ANOSIM p
genotype	Bray-Curtis	0,512179	<0,001	0,813667	<0,001
	Jaccard	0,363325	<0,001	0,894556	<0,001
	Weighted UniFrac	0,589345	<0,001	0,835556	<0,001
	Unweighted UniFrac	0,42598	<0,001	0,814667	<0,001

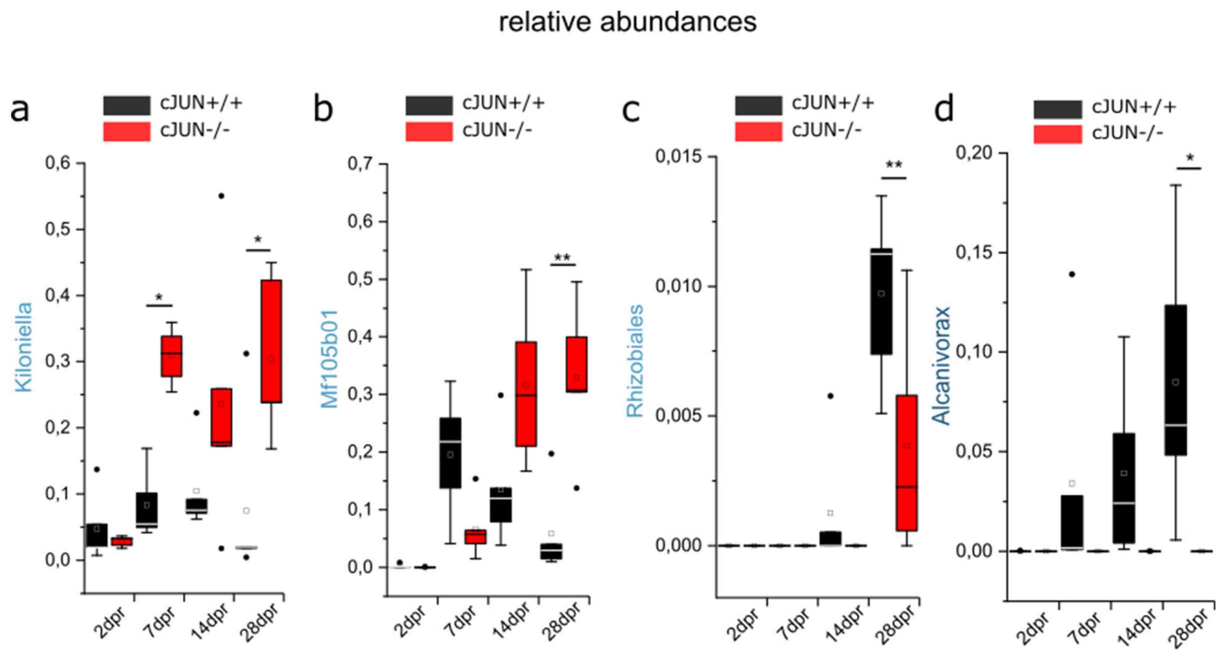

Figure S8. **Temporal dynamics of differentially abundant bacterial taxa in *cJUN*$^{-/-}$ and *cJUN*$^{+/+}$ polyps**. Relative abundances of specific bacterial taxa over time (2, 7, 14, and 28 days post-recolonization [dpr]) in cJUN knockout (red) and wildtype (black) polyps. **(a)** Kiloniella (Alphaproteobacteria), **(b)** Mf105b01 (Alphaproteobacteria), **(c)** Rhizobiales (Alphaproteobacteria), and **(d)** Alcanivorax (Gammaproteobacteria) show distinct colonization patterns between genotypes. Significant differences between groups at specific time points are indicated (* p <= 0.05, ** p <= 0.01, *** p <= 0.001; ANCOM). N = 5 polyps / genotype. Box plots indicate median (middle line), interquartile range (box), and range (whiskers), as well as outliers (single points).
