# Supplementary material for: c-JUN controls microbial colonization via selective phagocytosis in the sea anemone Nematostella
Source: Nat Commun. 2026 Jul 10;17:6087. doi: 10.1038/s41467-026-75511-w (PMC13354778; doi:10.1038/s41467-026-75511-w)
Supplement: Supplementary file 2 — Description of Additional Supplementary Files [file 41467_2026_75511_MOESM2_ESM.pdf]

Description of Additional Supplementary Files

Supplementary Movie. Localization and movement of nematosomes inside an adult *Nematostella vectensis* polyp, with focus on the tentacle region and mouth area. Freely moving nematosomes can be observed swimming within the gastric cavity.

Supplementary Data 1. All proteins identified from proteomic analysis. Nematosomes challenged with the native isolate NJ1, the non-native isolate Hal281, and non-bacterial challenge with a total number of 2,676 proteins listed with the Uniprot database Accession number and Uniprot database Annotation. This supplementary data is associated to Fig. 3.

Supplementary Data 2. KEGG pathway enrichment analysis of differentially abundant proteins identified across the indicated protein clusters. Significantly enriched KEGG pathways are shown for each cluster, including pathway annotation, number and percentage of proteins associated with each pathway, enrichment p-values, corresponding protein IDs, background population statistics, and fold enrichment values. Only pathways with significant enrichment are displayed. Pathways related to oxidative phosphorylation, ribosomal function, amino acid biosynthesis, carbon metabolism, phagosome formation, and general metabolic processes were among the most prominently enriched categories across clusters. Fold enrichment indicates the relative overrepresentation of pathway-associated proteins compared to the background proteome.

Supplementary Data 3. Identified proteins grouped into clusters based on ANOVA multiple-sample testing with a permutation-based false discovery rate (FDR) of 0.01. Clusters were categorized as follows: Cluster 1, proteins with high abundance exclusively in the native bacterial challenge; Cluster 2, proteins with high abundance in both native and foreign bacterial challenges; Cluster 3, proteins with high abundance in the native bacterial challenge and untreated control; Cluster 4, proteins with high abundance exclusively in the foreign bacterial challenge; Cluster 5, proteins with high abundance in the foreign bacterial challenge and untreated control; and Cluster 6, proteins with high abundance exclusively in the untreated control. Within each cluster, proteins are annotated according to UniProt accession number, UniProt annotation, KEGG annotation based on BlastKOALA analysis, and STRING database annotation.
