# Supplementary material for: c-JUN controls microbial colonization via selective phagocytosis in the sea anemone Nematostella
Source: Nat Commun. 2026 Jul 10;17:6087. doi: 10.1038/s41467-026-75511-w (PMC13354778; doi:10.1038/s41467-026-75511-w)
Supplement: Supplementary file 7 — Transparent Peer Review file [file 41467_2026_75511_MOESM7_ESM.pdf]

nature portfolio

Peer Review File

c-JUN controls microbial colonization via selective phagocytosis in the sea anemone Nematostella

Corresponding Author: Professor Sebastian Fraune

This file contains all reviewer reports in order by version, followed by all author rebuttals in order by version.

Version 0:

Reviewer comments:

Reviewer #1

(Remarks to the Author)
Summary
This study investigates the mechanism by which Nematostella selects certain bacteria for its microbiome and excludes others. Specifically, the study demonstrates a role for the nematosomes in regulating the composition of the microbiome by showing that “foreign” bacteria are phagocytosed and degraded by lysosomes in the nematosomes while “native” bacteria are not. Using CRISPR/Cas9 genome editing, the authors further demonstrate that knockout of cJun resulted in a reduced abundance of nematosomes and an increase in colonization of sea anemones by foreign bacteria. The main conclusion of the work is that adaptive immunity (i.e., the ability of the animal to respond in different ways to different bacteria) is more ancient than we once thought. This is an interesting study on the function of nematosomes that integrates multiple different types of data. While I appreciate the amount of work that went into this study, I find the conclusions to be a bit unfounded as they are only indirectly supported by the data. Furthermore, I am not convinced this study has really told us anything new about the evolution of immunity. I have outlined below several additional concerns – both general and specific – about the presentation of the data and the conclusions drawn from it. I encourage the authors to consider revising their work to address these points.

General comments
1. A main conclusion of this paper is that Nematostella selects some bacteria for its microbiome and excludes others. This conclusion is largely based on evidence of a greater relative abundance of bacteria in the phagocytes of nematosomes from animals inoculated with foreign bacteria vs native bacteria. However, to claim that nematosomes are “selective”, the authors would need to demonstrate that foreign bacteria are preferentially phagocytosed when the animals are presented with many types of bacteria, rather than in a mono-association experiment as they have done. The appropriate experiment would be to spike their inoculum during recolonization of WT and cJun KO animals with “foreign” bacteria and show the foreign bacteria are engulfed by the nematosomes in WT animals and not in KO animals. Indeed, the authors do not provide any direct evidence of variable nematosome abundance or behavior during recolonization experiments in WT vs KO animals, yet they claim the nematosomes are responsible for modulating the composition of the microbiome. While they do show that the abundance of nematosomes is different at a single time in the life of animals with and without a functional copy of cJun, it’s not possible to draw conclusions about the role of nematosomes during recolonization if the nematosomes were not actually studied during recolonization. I consider this to be a significant weakness of the study.

2. The strains of bacteria chosen for this study are poorly justified. First, it’s unclear how the authors define “foreign” bacteria – are they incapable of colonizing healthy Nematostella under wildtype conditions? Do they kill the animals or otherwise cause some kind of negative outcome when they do colonize? A major implication of the work is that foreign bacteria have some negative health impact on the animal and, thus, that the animal should want to eliminate them, but this is never demonstrated. Indeed, in Fig 2E the authors imply that the lower overall concentration of “foreign” bacteria is evidence that they are not as successful at colonizing the animals but the authors don’t justify how they conclude that overall concentration of bacteria is a good predictor of “successful” colonization. What if the “foreign” bacteria are just better at providing whatever service they provide to the sea anemones so they can exist at lower abundance in the animal? The authors cite 2-3 other studies on Nematostella which have demonstrated phagocytosis of E. coli and Vibrio coralliilyticus by the nematosomes – are those bacteria considered native or foreign? Do they have negative health outcomes? If they are foreign, then its not really clear to me that we learned anything new from this inoculation experiment since it has already been demonstrated that nematosomes can engulf foreign bacteria.

3. The evidence that phagocytosis of foreign bacteria by nematosomes is homologous to an “immune” reaction in other animals is fairly weak. The authors imply that the TLR/NFkB pathway is involved in phagocytosis of foreign bacteria, but these proteins do not appear to have been investigated in any way in this study. Indeed, the papers cited in this manuscript seem to indicate that TLR/NFkB are expressed in the nematocytes of the nematosomes, not the phagocytes. Furthermore, the ability of TLR to activate NFkB seems to have been studied only in cell culture, not in an actual sea anemone. The authors need to justify how they think TLR/NFkB could be involved in the phagocytosis and digestion of foreign bacteria if they are not even expressed in the phagocyte. The idea that this study challenges the classic dichotomy of innate vs adaptive immunity also seems oversold since the authors cite several other studies that have already found this result in other non-bilaterian taxa (e.g., sponges).

4. The cJun KO study is intriguing and the fact that the authors observed fewer nematosomes in cJun KO animals than in WT animals is a compelling and truly new piece of information about the biology of nematosomes, but this phenotype is insufficiently characterized. Are there negative health outcomes for animals that lack nematosomes? Is cJun expressed in the mesenteries (where the nematosomes are made)? If cJun controls the abundance of nematosomes and cJun is upregulated by inoculation with bacteria, one would expect there might be more nematosomes present over time during the recolonization experiments; this does not seem to have been measured. It is also imperative for the authors to demonstrate that nematosome abundance is low throughout recolonization experiments in the cJun KO animals, rather than at a single time point. Furthermore, while the authors show that the cJun KO animals experienced recolonization by different bacteria than WTs, there is no mention of health outcomes on the animals. Did they become sick after recolonization without nematosomes? If not, is there really any relevant effect here? This phenotype needs to be investigated more thoroughly.

Specific comments:

Introduction - The authors should provide some background about cJun in the introduction. The knockout of this gene is not justified in the intro and thus it is not clear what the expected phenotype would be.
Fig 2A - shows wildtype animals (with an intact cJun locus) engulf few native Vibrio and have few lysosomes and engulf many foreign Vibrio and have many lysosomes. By contrast, Figure 5A shows animals with an intact cJun locus engulf few foreign Vibrio and have lots of lysosomes. I’m having a hard time reconciling these results.
Fig 5C - It’s not clear what “lysosomes/nematosome (%)” means. Also, the caption suggests what was measured was “lysosomal area,” which is not explained. Several places throughout the manuscript the authors claim to have measured lysosome “activity” but this, again, is not explained. The behavior of the lysosomes is a big part of the story here and the methods used to investigate them are not clear or well-justified.
Fig 6 is not accessible to people outside of microbiome research. The metrics presented on the y-axes are not explained in enough detail for a reader to be able to determine whether the results match their expectations.
Throughout – the authors should be clear about how they are using the term “efficiency” as they seem to be reporting presence/absence or abundance of bacteria/lysosomes rather than directly measuring their activity or behavior.
Line 75 – remove “all” – it’s not possible to be sister to only some bilaterians.
Line 376 – proliferation should be abundance.
Line 497 – nematosomes are not cells, they are clusters of cells
Line 509 – the authors should be clear that this was a study of exogenously expressed TLR in mammalian cell culture. This protein may activate the same response in mammals as its mammalian homolog but that is not the same as activating the same response in cnidarians.

Reviewer #2

(Remarks to the Author)
Kaya and colleagues here present evidence that the Nematostella vectensis innate immune system selectively recognizes and destroys non-native bacteria through nematostome phagocytosis. The cJUN transcription factor is demonstrated to regulate the proliferation and lysosomal activity of the nematostomes, and thus to be integral to the structure of the microbiome. By utilizing a combination of methodologies from a new phagocytosis assay, proteomics, microbiome sequencing, and CRISPR/Cas9 knockouts, they gained a wholistic understanding of the mechanisms involved. As a sidenote, I really enjoyed the video! I found this paper intriguing and thorough, and recommend its publication with the following minor revisions.

Suggested minor revisions:
Line 94: It would be helpful to explain what the Hoechst and LysoTracker are staining in this image.
Line 98: It’s currently unclear why Figure 1C supports this statement, but if you explain the LysoTracker stain above that will help your reader understand
Line 106: I think the Figure 1D reference belongs in the sentence before this one, where the nematosomes packed into egg clutches is discussed.
Line 116-117: I would reverse the causal relationship – I think you are seeing that the selective phagocytosis of the nematostomes affects the composition of the microbiome, not the reverse as is implied in this sentence
Line 135: A better description of the bacterial isolates would be helpful. You mention later that these are Vibrios, but that should be included here as well. It would also be helpful to describe what Vibrios they are – are all 5 thought to be the same species? How similar are they phylogenetically? I think the mono-association section may have originally come first, and you have some description of these isolates there in lines 172-174. I would suggest moving that sentence here, but also addressing my other questions.

Throughout: You go back and forth between using a comma and a period as the decimal place. Either is fine, but it should be consistent throughout – it's in figures, legends, and the text in both ways. (example, compare line 136 and line 170)
Line 141: You say during bacterial staining here, but have provided no information about what staining was done. Please clarify.
Line 269: A brief overview of these parameters should be included here
Line 296: I'm unclear why 2B/C and 5B/C are labeled as percentages in the figure and in the legend. They appear to be counts? The numerical scale on the axes also does not appear to be a percentage. If this is actually a percentage then further information as to percent of what needs to be provided.
Line 301: I would reference the schematics in Figures 2 and 5 in the methods as well.
Line 310: Do you have a citation to support that Vibrios are the main colonizers of Nematostella? Is that in the wild or in the lab?
Line 360: I think it would be much more informative to label the clusters in the figure rather than list these cluster assignments in the legend.
Line 365: I really appreciated this diagram – nice job! I was a bit unclear on the grey boxes – are those proteins that weren't enriched in either treatment? I'd make that clearer.
Line 405: I am really surprised that polyp length here is not significant – are you sure? Regardless, I think you need to include your stats not just for significant results but also for those analyses that did not produce significance. This could be in a supplemental table or just in the legend or the text, but you should include the t-value and the df, as well as the p-value, for all analyses you did (so not just Fig 4 but also Fig 2, 5, 6).
Line 440: The color code in the lower left box of A is almost illegible, even when zoom is increased
Line 443: It would be good to discuss some of the strain differences you see in 5B, 2C, etc, somewhere in the paper.

Very minor revisions:
Line 53: into the phagosome
Lines 82-84: Please rework this sentence for greater clarity.
Line 92: represents
Line 93: with a blue
Line 106: Maybe include, "However, there has not yet been any direct evidence…"
Line 108: Here, we show that nematostomes…
Line 111-112: linked to a phagosomal pathway
Line 117: strong evidence that
Line 127: I think separated might be a better word here than organized
Line 136: I don't think you need the /mL for your OD600 – that is implied.
Line 138: in the dark … bacteria were centrifuged
Line 147: nematostomes were stained
Line 150: mounted on a slide
Line 151: Images were taken…
Line 169, 181, 277: prior to _____
Line 178: I think instead of succession here you mean success?
Lines 182-184: Please rework these two sentences for better clarity.
Line 186: and centrifuged at 5000
Line 186-187: I would either remove "to the bottom of the tube" or change to "to collect in the bottom of the tube"
Line 189: vortexing every 15 min
Line 189-190: were snap-frozen at
Line 197: mixed by pipetting … kept on the shaker
Line 276: …and fasttree2 was used to construct the phylogeny.
Line 277: per sample prior
Line 287: Most of your results section is in past tense, make sure you are consistent (here replace are with were)
Line 326: lysosomal activity
Line 345: host metabolism,
Line 347-349: Proteins belonging to… F-actin are especially increased.
Line 356-358: Please rework this sentence for clarity
Line 379: identified a cJUN
Line 384: As the central hub for ___________, cJUN…
Line 402-403. The sentences that start "F2 with…" and "Additional predicted…" are both fragments – please edit to make complete sentences.
Line 421-422: Maybe "the phagocytosis assay developed above"
Line 428: Hal281 did not result in the activation
Line 436-437: Please rework the clause starting "suggesting even" for clarity
Line 543: However, symbionts also rely
Line 565: don't need both "In addition" and "also", choose one

Version 1:

Reviewer comments:

Reviewer #1

(Remarks to the Author)
The authors have done a very good job with this revision and I consider the manuscript significantly improved and ready for publication. One small point, regarding comment 4.3: given that cJun is expressed in several tissues (not just nematosomes - Fig 4a) and given that the knockout is not tissue-specific, the authors should use caution in attributing the decreases in offspring fitness to maternal provisioning of nematosomes (Line 266-281 results). There could be other (unmeasured) effects of cJun KO that impacted offspring survival. I have no other concerns - nice job with the revisions!

Reviewer #2

(Remarks to the Author)
You have done a nice job of addressing all the comments raised in the original review. I was happy with the revisions and think this paper is ready to be published.

Open Access This Peer Review File is licensed under a Creative Commons Attribution 4.0 International License, which permits use, sharing, adaptation, distribution and reproduction in any medium or format, as long as you give appropriate credit to the original author(s) and the source, provide a link to the Creative Commons license, and indicate if changes were made.
In cases where reviewers are anonymous, credit should be given to 'Anonymous Referee' and the source.
The images or other third party material in this Peer Review File are included in the article's Creative Commons license, unless indicated otherwise in a credit line to the material. If material is not included in the article's Creative Commons license and your intended use is not permitted by statutory regulation or exceeds the permitted use, you will need to obtain permission directly from the copyright holder.
To view a copy of this license, visit https://creativecommons.org/licenses/by/4.0/

Reviewer #1 (Remarks to the Author):

Summary

This study investigates the mechanism by which Nematostella selects certain bacteria for its microbiome and excludes others. Specifically, the study demonstrates a role for the nematosomes in regulating the composition of the microbiome by showing that "foreign" bacteria are phagocytosed and degraded by lysosomes in the nematosomes while "native" bacteria are not. Using CRISPR/Cas9 genome editing, the authors further demonstrate that knockout of cJun resulted in a reduced abundance of nematosomes and an increase in colonization of sea anemones by foreign bacteria. The main conclusion of the work is that adaptive immunity (i.e., the ability of the animal to respond in different ways to different bacteria) is more ancient than we once thought. This is an interesting study on the function of nematosomes that integrates multiple different types of data. While I appreciate the amount of work that went into this study, I find the conclusions to be a bit unfounded as they are only indirectly supported by the data. Furthermore, I am not convinced this study has really told us anything new about the evolution of immunity. I have outlined below several additional concerns – both general and specific – about the presentation of the data and the conclusions drawn from it. I encourage the authors to consider revising their work to address these points.

We thank the reviewer for the careful evaluation of our manuscript and the thoughtful and constructive feedback. We appreciate the reviewer's recognition of the extensive experimental work performed in this study and the opportunity to further clarify several aspects of our interpretation and experimental design. In response to these comments, we have performed several new experiments and revised the manuscript to better justify our conclusions, clarify definitions and methodological descriptions, and moderate several interpretations where appropriate. Below we address each point in detail.

General comments

1.1. A main conclusion of this paper is that Nematostella selects some bacteria for its microbiome and excludes others. This conclusion is largely based on evidence of a greater relative abundance of bacteria in the phagocytes of nematosomes from animals inoculated with foreign bacteria vs native bacteria. However, to claim that nematosomes are "selective", the authors would need to demonstrate that foreign bacteria are preferentially phagocytosed when the animals are presented with many types of bacteria, rather than in a mono-association experiment as they have done.

Response: We thank the reviewer for raising the important point regarding the interpretation of selective phagocytosis. We agree that demonstrating differential uptake when animals are exposed to multiple bacterial strains simultaneously provides a stronger test for selective bacterial recognition. To address this concern, we have expanded our analysis and now include an experiment in which nematosomes were challenged with two bacterial isolates simultaneously. The relative contribution of each bacterium to the total engulfed bacterial signal was quantified and is now presented as stack bars representation in **Figure 5d**. This analysis allows a direct comparison of bacterial uptake within the same nematosomes under mixed bacterial exposure. The results show that nematosomes from wildtype animals preferentially engulf the foreign isolates compared to the native isolates, whereas this discrimination is lost in *cJUN*$^{-/-}$ animals, where both bacterial strains are engulfed at similar proportions. These results therefore provide additional evidence that nematosomes exhibit differential phagocytotic responses toward distinct bacterial isolates and that this discriminatory capacity depends on cJUN.

Line 337-342 (Results): *"To determine whether this difference in lysosomal activation by foreign and native Vibrio isolates leads to selective phagocytosis, we conducted a phagocytosis competition experiment in which we exposed nematosomes to both a native and a foreign isolate at the same concentration (Fig. 5d). This experiment showed that cJUN$^{+/+}$ nematosomes preferentially phagocytose foreign isolates at a phagocytosis rate of approximately 70%, whereas mutant nematosomes lose this preferential phagocytosis of foreign isolates and engulf both bacteria at a similar rate (Fig. 5d)."*

1.2. The appropriate experiment would be to spike their inoculum during recolonization of WT and cJun KO animals with "foreign" bacteria and show the foreign bacteria are engulfed by the nematosomes in WT animals and not in KO animals. Indeed, the authors do not provide any direct evidence of variable nematosome abundance or behavior during recolonization experiments in WT vs KO animals, yet they claim the nematosomes are responsible for modulating the composition of the microbiome. While they do show that the abundance of nematosomes is different at a single time in the life of animals with and without a functional copy of cJun, it's not possible to draw conclusions about the role of nematosomes during recolonization if the nematosomes were not actually studied during recolonization. I consider this to be a significant weakness of the study.

Response: We appreciate this thoughtful suggestion and agree that directly monitoring nematosomes abundance and behavior throughout recolonization experiments would provide valuable mechanistic insight into how nematosomes influence microbiome assembly.

However, such an experiment would require a substantially expanded experimental framework, including parallel long-term recolonization experiments in both *cJUN*$^{+/+}$ and *cJUN*$^{-/-}$ animals, repeated sampling across multiple time points, simultaneous quantification of nematosomes abundance, and microbiome profiling in matched individuals. In addition, direct visualization of nematosome-bacteria interactions during recolonization would require the establishment of live imaging approaches under gnotobiotic conditions. We consider this an important future direction, but one that expands beyond the scope of the present revision.

Importantly, the current study already provides several independent lines of evidences linking nematosomes to microbiome regulation: (i) *cJUN*$^{-/-}$ animals exhibit strongly reduced nematosomes abundance **(Figure 4f)**, (ii) mutant nematosomes lose differential bacterial recognition **(Figure 5a-d)**, (iii) non-native bacterial isolates recolonize mutant animals more successfully **(Figure 5e)**, and (iv) *cJUN*$^{-/-}$ animals develop an altered microbiome composition over time **(Figure 6d)**. Together, these findings support a functional relationship between nematosomes biology and host-associated microbiome structure.

To avoid overstating causality, we have revised the manuscript to clarify that our data support strong association between cJUN-dependent nematosomes function and microbiome composition, while direct real-time analyses during recolonization remain an important avenue for future.

Line 423-426 (Discussion): *"The observed correlation between phagocytosis efficiency and bacterial colonization rate supports the hypothesis that nematosomes act as selective gatekeepers of microbial establishment in N. vectensis. This correlation was functionally linked in cJUN$^{-/-}$ nematosomes that lost the ability to differentiate between foreign and native isolates in a competition phagocytosis assay."*

2.1 The strains of bacteria chosen for this study are poorly justified. First, it's unclear how the authors define "foreign" bacteria – are they incapable of colonizing healthy Nematostella under wildtype conditions?

Response: We thank the reviewer for this helpful comment and agree that the rationale for isolate selection and terminology required clearer explanation.

Information regarding all bacterial isolates used in this study was already included in Supplementary Table 1, which summarizes isolate identity, original host source, closest type strain assignment, sequence similarity, and relevant references. To improve accessibility for readers, we now refer to this table more explicitly already in the Methods section where the isolates are first mentioned. Specifically, isolates NJ1, NJ33, and NA11 were originally obtained from *Nematostella vectensis* and are therefore referred to as native host-associated isolates. In contrast, Hal025 and Hal281 were isolated from the marine sponge *Halichondira panicea* and are therefore described as non-native isolates. *Vibrio coralliilyticus*, however, is a pathogen obtained from *Pocillopora damicornis*. This terminology is not referring to the ability to colonize the host, as all isolates can colonize the host, but in different quantities.

"Table S1. ***Bacterial isolates used in this study and their taxonomic classification.*** *Overview of all bacterial isolates used for recolonization, phagocytosis, and pathogen challenge experiments in Nematostella vectensis. The table includes isolate origin (host organism), experimental role (native, foreign, or pathogen), available GenBank accession numbers, closest related type strains based on 16S rRNA gene sequence similarity, percentage sequence identity, corresponding ASV assignments according to Domin et al. 2023, and original references describing the isolates. Native isolates (NJ1, NJ33, and NA11) were originally isolated from N. vectensis, whereas foreign isolates (Hal025 and Hal281) originated from the sponge Halichondria panicea. Vibrio coralliilyticus was used as a pathogenic challenge strain in infection experiments obtained from Pocillopora damicornis."*

2.2 Do they kill the animals or otherwise cause some kind of negative outcome when they do colonize? A major implication of the work is that foreign bacteria have some negative health impact on the animal and, thus, that the animal should want to eliminate them, but this is never demonstrated. Indeed, in Fig 2E the authors imply that the lower overall concentration of "foreign" bacteria is evidence that they are not as successful at colonizing the animals but the authors don't justify how they conclude that overall concentration of bacteria is a good predictor of "successful" colonization. What if the "foreign" bacteria are just better at providing whatever service they provide to the sea anemones so they can exist at lower abundance in the animal?

<u>Response:</u> We further like to clarify that the term "non-native" or "foreign" does not imply pathogenicity. Rather these strains originate from a different host-associated microbial environment and are not typical members of the *Nematostella* microbiome. To distinguish colonization compatibility from pathogenicity, we additionally included host fitness and survival analyses in the revised manuscript. To detect subtle physiological consequences of altered colonization beyond survival, we first established a standardized morphology-based health scoring assay for *Nematostella* under laboratory conditions **(Supplementary Figure 3)**. These show that the tested native and foreign isolates did not induce mortality **(Supplementary Figure 1g, h)**, or any other detectable fitness disadvantage or advantage under tested condition **(Figure 5f, Supplementary Figure 6c)** whereas the established pathogen *Vibrio coralliilyticus* caused strong mortality **(Figure 4h, i, Supplementary Figure 1i, j)**.

2.3 The authors cite 2-3 other studies on Nematostella which have demonstrated phagocytosis of E. coli and Vibrio coralliilyticus by the nematosomes – are those bacteria considered native or foreign? Do they have negative health outcomes? If they are foreign, then its not really clear to me that we learned anything new from this inoculation experiment since it has already been demonstrated that nematosomes can engulf foreign bacteria.

<u>Response:</u> We appreciate the concern and would like to clarify that the novelty of our study is not the demonstration that phagocytosis itself represents an immune mechanism, as phagocytosis is a conserved defense process broadly present across metazoans. Rather, the key finding of our work is that nematosomes exhibit differential phagocytic response toward closely related bacterial isolates, accompanied by isolate-specific lysosomal activation and consequences for bacterial colonization success. We have revised the manuscript to emphasize this distinction more clearly.

Line 407-408 (Discussion): *"Our study provides strong evidence that nematosomes are key immune structures in N. vectensis, playing a critical role in microbial selection through differential phagocytosis."*

Line 526-528 (Discussion): *"These findings support the idea that fine-scale microbial recognition and differential host responses to closely related bacterial strains may represent a conserved feature of host-microbe interaction across diverse symbiotic systems"*

Together, these clarifications strengthen the rationale for the selected isolates and better define their biological context.

3. The evidence that phagocytosis of foreign bacteria by nematosomes is homologous to an "immune" reaction in other animals is fairly weak. The authors imply that the TLR/NFkB pathway is involved in phagocytosis of foreign bacteria, but these proteins do not appear to have been investigated in any way in this study. Indeed, the papers cited in this manuscript seem to indicate that TLR/NFkB are expressed in the nematocytes of the nematosomes, not the phagocytes. Furthermore, the ability of TLR to activate NFkB seems to have been studied only in cell culture, not in an actual sea anemone. The authors need to justify how they think TLR/NFkB could be involved in the phagocytosis and digestion of foreign bacteria if they are not even expressed in the phagocyte. The idea that this study challenges the classic dichotomy of innate vs adaptive immunity also seems oversold since the authors cite several other studies that have already found this result in other non-bilaterian taxa (e.g., sponges).

Response: We thank the reviewer for this thoughtful comment and agree that several conceptual distinctions required clearer wording. We approve that the potential involvement of TLR/NFkB signaling pathways in the nematosome-mediated bacterial recognition was not directly tested in the present study. Our intention was to place our findings within the broader context of previously described innate immune pathways in cnidarians. To avoid overstanding this point, we have revised the Discussion to explicitly state that the interaction between canonical immune signaling pathways (such as TLR/NFkB) and nematosome phagocytotic function remains hypothetical and represents an important direction for future work.

Line 418-421 (Discussion): *"In nematosomes of N. vectensis components of the TLR signaling pathway have previously been reported. However, whether these pathways directly regulate nematosome-mediated phagocytosis remain unknown and was not tested in the present study."*

We also agree that the previous wording regarding adaptive immunity was too strong. We have therefore revised Abstract and Discussion to avoid implying the presence of adaptive immunity in cnidarians. Instead, we now emphasize that our findings support the presence of sophisticated innate immune discrimination mechanism and suggest that microbial recognition specificity may have evolved early during animal evolution.

Line 43-44 (Abstract): "*Our findings support the idea that immune specificity can arise through repurposing deeply conserved pathways and may have deep evolutionary origin."*

Line 563-566 (Discussion): *"This study, therefore, positions cnidarians as valuable models for exploring the evolutionary origins of immune-microbe interactions and provides insights into how early metazoans may have developed mechanisms for microbial regulation through selective innate immunity in the absence of adaptive immunity."*

4.1 The cJun KO study is intriguing and the fact that the authors observed fewer nematosomes in cJun KO animals than in WT animals is a compelling and truly new piece of information about the biology of nematosomes, but this phenotype is insufficiently characterized. Are there negative health outcomes for animals that lack nematosomes?

Response: We thank the reviewer for recognizing the novelty and significance of the *cJUN*$^{-/-}$ phenotype. In response to this comment, we substantially expanded the phenotypic characterization *of cJUN*$^{-/-}$ animals in the revised manuscript.

To address the biological relevance of this phenotype, we added multiple fitness assays. We now show that *cJUN*$^{-/-}$ animals display increased susceptibility to infection with the pathogen *Vibrio coralliilyticus*, resulting in elevated mortality compared to WT polyps **(Figure 4h, i)**. These findings indicate that cJUN-dependent signaling contribute to host defense.

Line 282-297 (Results): "To determine whether the reduced nematosome abundance in cJUN$^{-/-}$ *animals affects host resilience to infection, we compared the survival of cJUN*$^{+/+}$ *and cJUN*$^{-/-}$ *polyps following exposure to the pathogenic bacterium V. coralliilyticus (Fig. 4h, i). Survival was monitored over nine days at two temperatures, 18 °C (Fig. 4h) and 30 °C (Fig. 4i), representing elevated stress conditions. Kaplan-Meier survival analysis revealed significant genotype-dependent differences in survival following pathogen exposure. While cJUN*$^{+/+}$ *polyps showed higher survival rates during infection, cJUN*$^{-/-}$ *animals exhibited a significant increased mortality. This effect became particularly pronounced under elevated temperature condition, where pathogen induced mortality progressed more rapidly in mutant animals compared to cJUN*$^{+/+}$ *polyps (Fig. 4i). In contrast, cJUN*$^{-/-}$ *animals without bacterial challenge, as well as animals exposed to native and foreign colonizer, showed unaffected survival rates (Supplementary Fig. 1g, h). Interestingly, GF animals, independent of genotype, exhibit higher mortality following infection with V. coralliilyticus than animals with an intact microbiome (Supplementary Fig. 1i, j), suggesting that the microbiome provides general protection against infection with V. coralliilyticus. These findings indicate that cJUN contributes to host defense against bacterial infection and suggest that the reduced nematosome abundance in cJUN*$^{-/-}$ *animals compromises the host's ability to control pathogenic bacteria."*

In addition, as altered bacterial colonization may produce sublethal effects not captured by survival alone, we established a standardized morphology-based health scoring assay for *Nematostella vectensis* **(Supplementary Figure 3)**. Using this framework, we observed reduced condition scores in *cJUN*$^{-/-}$ animals under specific recolonization conditions **(Figure 5f, Supplementary Figure 6c)**, indicating that the mutant phenotype affects host performance even in the absence of acute mortality.

Line 348-352 (Result): "To evaluate whether these altered colonization dynamics affect host health, we assessed polyp condition scores following the bacterial mono-associations (Fig. 5f, Supplementary Fig. 3, 6c). Polyp health scoring revealed significant effects of both genotype and bacterial treatment, with mutant animals displaying reduced health under specific bacterial challenges compared to WT animals."

4.2 Is cJun expressed in the mesenteries (where the nematosomes are made)?

Response: Regarding tissue localization, cJUN expression profiles presented in Figure 4a support strong abundance in nematosomes and slight abundance in mesenterial tissues, consistent with the mesenteries being the site of nematosomes formation.

4.3 If cJun controls the abundance of nematosomes and cJun is upregulated by inoculation with bacteria, one would expect there might be more nematosomes present over time during the recolonization experiments; this does not seem to have been measured. It is also imperative for the authors to demonstrate that nematosome abundance is low throughout recolonization experiments in the cJun KO animals, rather than at a single time point. Furthermore, while the authors show that the cJun KO animals experienced recolonization by different bacteria than WTs, there is no mention of health outcomes on the animals. Did they become sick after recolonization without nematosomes? If not, is there really any relevant effect here? This phenotype needs to be investigated more thoroughly.

Response: We appreciate the reviewer's interest in directly monitoring nematosome dynamics during recolonization. As outlined above, a longitudinal analysis of nematosomes abundance and behavior

throughout recolonization would require a substantially expanded experimental framework, including repeated parallel sampling of *cJUN*$^{+/+}$ and *cJUN*$^{-/-}$ animals across multiple time points, matched microbiome profiling, and continuous imaging of nematosome release and activity under controlled gnotobiotic conditions. We agree that such experiments would provide valuable mechanistic insight, but they are currently beyond the scope of the present revision.

To address the reviewer's broader request for a comprehensive characterization of the mutant phenotype, we instead expanded the biological analysis of *cJUN*$^{-/-}$ animals in a complementary direction. Specifically, we investigated reproductive and maternal consequences of reduced nematosome abundance. We show that egg packages derived from *cJUN*$^{-/-}$ females contain fewer maternally transferred nematosomes compared with wildtype animals. In addition, offspring originating from mutant mothers exhibit reduced long-term survival **(Supplementary Figure 1)**. These new data indicate that cJUN-dependent nematosome regulation has consequences beyond adult recolonization and may influence host fitness across generations.

<u>Line 266-281 (Results):</u> *"Since nematosomes are maternally deposited into the egg packages, we next asked whether the cJUN mutation also affects the abundance of nematosomes transferred to developing embryos and the fitness of the corresponding offspring. Examination of egg packages released from adult female polyps revealed the presence of numerous nematosomes embedded within the gelatinous matrix surrounding the embryos (Supplementary Fig. 1a-c). Consistent with the reduced nematosomes abundance observed in female adult mutants, egg packages derived from cJUN$^{-/-}$ animals contained significantly fewer nematosomes compared to those from cJUN$^{+/+}$ female animals (Supplementary Fig. 1d, e). To assess the functional consequences of reduced maternal nematosome transfer, we monitored larval survival over an extended developmental period (Supplementary Fig. 1f). Offspring originated from cJUN$^{-/-}$ female polyps exhibited a significant higher decline in survival over time, compared to offspring from cJUN$^{+/+}$ mothers (Supplementary Fig. 1f). The survival rate of the offspring depended on the mother's genotype and not on the offspring's genotype, since cJUN$^{+/+}$ oocytes fertilized with cJUN$^{-/-}$ sperm, which produced also heterozygous F1 offspring, exhibit a similarly high survival rate as the offspring from a cJUN$^{+/+}$ crossing (Supplementary Fig. 1f). These findings suggest that maternal provisioning of nematosomes contributes to long-term offspring survival and early-life protection."*

Specific comments:

Introduction - The authors should provide some background about cJun in the introduction. The knockout of this gene is not justified in the intro and thus it is not clear what the expected phenotype would be.

<u>Response:</u> We thank the reviewer for this helpful suggestion and agree that additional background on cJUN improves the rational for the knockout experiments. In the revised Introduction, we now introduce cJUN as an evolutionarily conserved AP-1 family transcription factor involved in immune regulation, phagocytosis-related signaling, and cell proliferation across metazoans. We further note that the *Nematostella* cJUN ortholog (NVE21090) was identified from previous transcriptomic datasets as being responsive to bacterial recolonization **(Supplementary Figure 2)** and enriched in nematosomes-associated tissues.

<u>Line 110-116 (Introduction)</u>: *"Previous transcriptome analyses have shown that a cJUN ortholog in N. vectensis responds to bacterial recolonization, making it a promising candidate for regulating nematosome function (Supplementary Fig.2). cJUN is an evolutionarily conserved transcription factor that activate immune genes, regulates phagocytosis, and is involved in cell proliferation. As the central hub, cJUN integrates signaling information of various pathways, including ERK and JNK signaling. In the cnidarian Hydra TLR signaling via MyD88 activates JNK signaling following immune stimulation and in vertebrates cJUN regulates macrophage activation."*

Fig 2A - shows wildtype animals (with an intact cJun locus) engulf few native Vibrio and have few lysosomes and engulf many foreign Vibrio and have many lysosomes. By

contrast, Figure 5A shows animals with an intact cJun locus engulf few foreign Vibrio and have lots of lysosomes. I'm having a hard time reconciling these results.

Response: We appreciate the reviewer for carefully noting this point. We agree that the originally selected representative image in Figure 5a did not optimally reflect the quantitative data shown in the corresponding data and could therefore lead to confusion when compared to Figure 2a.

To address this, we replaced Figure 5a with more representative confocal image that better reflects the overall experimental outcome and is more consistent with the quantified bacterial uptake and lysosomal response shown in Figure 5b, c. We further clarify in the revised legend that all microscopy panels are representative examples, whereas quantitative conclusions are based on the associated statistical analyses.

Line 138-139: "Representative confocal images of nematosomes challenged with NJ1 (native colonizer) and Hal281 (foreign colonizer)."

Fig 5C - It's not clear what "lysosomes/nematosome (%)" means. Also, the caption suggests what was measured was "lysosomal area," which is not explained. Several places throughout the manuscript the authors claim to have measured lysosome "activity" but this, again, is not explained. The behavior of the lysosomes is a big part of the story here and the methods used to investigate them are not clear or well-justified.

Response: We thank the reviewer for identifying the need for clearer terminology. We have revised the manuscript, figures, and legends to explicitly state that this metric represents the LysoTracker-positive area normalized to total nematosome area (%), as quantified by image analysis. This is also described in the Methods (Phagocytosis assay) section.

Line 627-630 (Methods): "For the quantification of lysosomal positive area, the LysoTracker-positive area was measured and normalized to the total nematosome area using image analysis in ImageJ Fiji. The resulting values are presented as the percentage of LysoTracker-positive area relative to the total nematosome area (%)."

We further clarified that LysoTracker staining was used as a proxy for acidic lysosomal compartments and therefore reflects relative lysosomal abundance / activation rather than direct enzymatic activity measurements. Accordingly, we have replaced ambiguous wording such as "lysosomal activity" where appropriate with more precise terminology including "relative lysosomal area" or "LysoTracker-positive lysosomal signal".

*Line 320: "**c** LysoTracker-positive lysosomal area relative to total nematosome area (%)."*

Fig 6 is not accessible to people outside of microbiome research. The metrics presented on the y-axes are not explained in enough detail for a reader to be able to determine whether the results match their expectations.

Response: We thank reviewer for this valuable comment. We agree that the microbiome diversity metrics required clearer explanation for broader readership.

We have therefore expanded the figure legend to briefly define the present metrics. Specifically, we now explain that Faith's Phylogenetic Diversity reflects phylogenetic richness, evenness reflects the distribution of taxa across the community, and Weighted UniFrac measures phylogenetically informed community differences while accounting for relative abundance.

***Line 380-387:** "**a** Faith's phylogenetic diversity, reflecting the evolutionary richness of bacterial communities. **b** Pielou's evenness, indicating how evenly bacterial taxa are distributed within each community. (…). **c** Weighted UniFrac distance comparisons between microbial communities of cJUN$^{+/+}$ and cJUN$^{-/-}$ polyps over time. Weighted UniFrac considers both bacterial relatedness and relative abundance, with larger distances indicating greater differences between microbial communities."*

Throughout – the authors should be clear about how they are using the term "efficiency" as they seem to be reporting presence/absence or abundance of bacteria/lysosomes rather than directly measuring their activity or behavior.

Response: We agree that the term "efficiency" was too imprecise in several context. Throughout the revised manuscript, we replaced this wording with some specific descriptions such as:

Bacterial engulfment level, relative bacterial uptake, colonization success, relative lysosomal area

Line 75 – remove "all" – it's not possible to be sister to only some bilaterians.

Response: Corrected as suggested.

Line 376 – proliferation should be abundance.

Response: Corrected as suggested

Line 497 – nematosomes are not cells, they are clusters of cells

Response: Corrected as suggested

Line 509 – the authors should be clear that this was a study of exogenously expressed TLR in mammalian cell culture. This protein may activate the same response in mammals as its mammalian homolog but that is not the same as activating the same response in cnidarians.

Response: We thank the reviewer for this important clarification and agree that the previous wording overstated the experimental evidence regarding TLR/NF-κB signaling in cnidarians. In response, we substantially revised this section of the Discussion to clearly distinguish previously published heterologous expression studies from direct functional evidence in Nematostella. Specifically, we removed statements implying that nematosome-associated TLR signaling directly activates NF-κB-mediated bacterial recognition pathways in vivo. Instead, we now state more cautiously that components of the TLR signaling pathway have been reported in nematosomes, while emphasizing that their direct involvement in nematosome-mediated phagocytosis remains unknown and was not tested in the present study.

Line 418-421 (Discussion): "In nematosomes of N. vectensis components of the TLR signaling pathway have previously been reported. However, whether these pathways directly regulate nematosome-mediated phagocytosis remain unknown and was not tested in the present study."

We further retained the Hydra MyD88 example only as broader evolutionary context linking TLR-associated signaling to cJUN-dependent microbiome regulation in cnidarians, rather than as direct mechanistic evidence for nematosome function.

Reviewer #2 (Remarks to the Author):

Kaya and colleagues here present evidence that the Nematostella vectensis innate immune system selectively recognizes and destroys non-native bacteria through nematostome phagocytosis. The cJUN transcription factor is demonstrated to regulate the proliferation and lysosomal activity of the nematostomes, and thus to be integral to the structure of the microbiome. By utilizing a combination of methodologies from a new phagocytosis assay, proteomics, microbiome sequencing, and CRISPR/Cas9 knockouts, they gained a wholistic understanding of the mechanisms involved. As a sidenote, I really enjoyed the video! I found this paper intriguing and thorough, and recommend its publication with the following minor revisions.

We thank the Reviewer #2 for the positive assessment of our work and for the thoughtful and constructive suggestions. We greatly appreciate the reviewer's recognition of the multidisciplinary approach of this study and are pleased that the supplementary video was enjoyable. We have carefully revised the manuscript according to all suggestions, which substantially improved clarity and accessibility of the manuscript.

Suggested minor revisions:

Line 94: It would be helpful to explain what the Hoechst and LysoTracker are staining in this image.

Response: We have revised the corresponding figure legend and the text to clarify that Hoechst strains nuclei (DNA), whereas LysoTracker labels acidic lysosomal compartments.

Line 97-98: "Confocal image of a nematosome stained with Hoechst (blue), labeling nuclei, and LysoTracker (red), labeling acidic lysosomal compartments. Scalebar represent 10 µm"

Line 98: It's currently unclear why Figure 1C supports this statement, but if you explain the LysoTracker stain above that will help your reader understand

Response: We thank the reviewer for noting this lack of clarity. By clarifying the LysoTracker staining as a marker for lysosomal compartments, the relevance of Figure 1c to support the phagocytic function of nematosomes is now more explicit in the revised text.

Line 106: I think the Figure 1D reference belongs in the sentence before this one, where the nematosomes packed into egg clutches is discussed.

Response: Done. We have moved the figure citation accordingly.

Line 116-117: I would reverse the causal relationship – I think you are seeing that the selective phagocytosis of the nematostomes affects the composition of the microbiome, not the reverse as is implied in this sentence

Response: We thank the reviewer for this important point and agree with the revised interpretation. The sentence has been rewritten to clarify that selective phagocytosis by nematosomes likely contributes to shaping microbiome composition, rather than implying the reverse causal direction.

Line 123-126 (Introduction): "These cJUN$^{-/-}$ polyps exhibit a reduced fitness after Vibrio coralliilyticus (V. coralliilyticus) and are colonized by an altered microbiome, demonstrating a causal relationship between the selective phagocytosis of the nematosomes and microbiome colonization."

Line 135: A better description of the bacterial isolates would be helpful. You mention later that these are Vibrios, but that should be included here as well. It would also be helpful to describe what Vibrios they are – are all 5 thought to be the same species? How similar are they phylogenetically? I think the mono-association section may have originally come first, and you have some description of these isolates there in lines 172-174. I would suggest moving that sentence here, but also addressing my other questions.

Response: We thank reviewer for this suggestion. We have expanded the description of the bacterial isolates in the revised manuscript and now explicitly state their host origin and ecological context and the role in this study (native, foreign, pathogen). In addition, Supplementary Table 1 provides taxonomic affiliation, closest type strain assignment, sequence similarity, and references for all isolates used in this study.

Throughout: You go back and forth between using a comma and a period as the decimal place. Either is fine, but it should be consistent throughout – it's in figures, legends, and the text in both ways. (example, compare line 136 and line 170)

Response: We thank the reviewer for identifying this inconsistency. Decimal notation has now been standardized throughout the manuscript, figures, and legends.

Line 141: You say during bacterial staining here, but have provided no information about what staining was done. Please clarify.

Response: We have revised the Methods section to specify that bacterial cells were fluorescently labeled using BacLight dyes prior to the phagocytotic assay.

Line 611-612 (Methods): "1 mL of bacteria was centrifuged and diluted with sterile 16 ‰ NM prior staining with BacLight (0.1 µM, Thermo Fisher) for 15 min in the dark and room temperature."

Line 269: A brief overview of these parameters should be included here

Response: We thank the reviewer for pointing this out. We have revised the corresponding Methods section to provide a clearer brief overview of the electron microscopy preparation, including sample fixation, dehydration, and coating, to improve reproducibility and reader accessibility.

Line 779-788 (Methods): "Samples were first fixed for 1 h at RT in 4 % glutaraldehyde prepared in 0.1 M Na cacodylate buffer, followed by three washes of 10 min each in 0.1 M cacodylate buffer. A second fixation step was then carried out for 1 h in the dark at 25 °C using 2 % osmium tetraxide (in 0.2 M cacodylate buffer). After fixation, samples were washed three times for 10 min each in 0.1 M cacodylate buffer. Dehydration was performed through a graded ethanol series beginning with 30 % ethanol for 10 min, followed by 50 % ethanol for 2 x 15 min, 70 % ethanol for 15 min, and an additional 70 % ethanol overnight, all at RT. On the following day, dehydration was continued with 80 % ethanol for 2 x 15 min, 90 % ethanol for 2 x 15min, 96 % ethanol for 2x 15 min, and finally 100 % ethanol for 2 x 15 min, all at RT. Samples were subsequently critical point dried, mounted, and sputter coated prior to imaging. Acquired images were analyzed using ImageJ Fiji "

Line 296: I'm unclear why 2B/C and 5B/C are labeled as percentages in the figure and in the legend. They appear to be counts? The numerical scale on the axes also does not appear to be a percentage. If this is actually a percentage then further information as to percent of what needs to be provided.

Response: We thank the reviewer for noting this ambiguity. We revised the legends to clearly state that these values represent signal area normalized to total nematosome area (%), rather than raw counts. Additionally, we included in the Methods (Phagocytosis Assay) the following line:

Line 627-630: "For the quantification of lysosomal positive area, the LysoTracker-positive area was measured and normalized to the total nematosome area using image analysis in ImageJ Fiji. The resulting values are presented as the percentage of LysoTracker-positive area relative to the total nematosome area (%)."

Line 301: I would reference the schematics in Figures 2 and 5 in the methods as well.

Response: Done. Appropriate figure references were added to the Methods section.

Line 645-647: "Antibiotic treatment (AB treatment) approaches were adapted from the established protocol for generating germfree Hydra polyps and further adapted for Nematostella (Fig. 2d, Supplementary Fig. 6a)."

Line 310: Do you have a citation to support that Vibrios are the main colonizers of Nematostella? Is that in the wild or in the lab?

Response: We thank the reviewer for this suggestion. Appropriate reference supporting the prevalence of *Vibrio*-related taxa in *Nematostella vectensis* microbiome have been added and clarified.

Mortzfeld BM, Urbanski S, Reitzel AM, Kunzel S, Technau U, Fraune S. Response of bacterial colonization in Nematostella vectensis to development, environment and biogeography. Environ Microbiol 18, 1764–1781 (2016).

Baldassarre L, Reitzel AM, Fraune S. Genotype-environment interactions determine microbiota plasticity in the sea anemone Nematostella vectensis. PLoS Biol 21, e3001726 (2023).

Line 360: I think it would be much more informative to label the clusters in the figure rather than list these cluster assignments in the legend.

Response: Done. Cluster identities are now indicated more clearly in the revised figure.

Line 365: I really appreciated this diagram – nice job! I was a bit unclear on the grey boxes – are those proteins that weren't enriched in either treatment? I'd make that clearer.

Response: We thank the reviewer for this helpful comment. The legend has been revised to clarify that grey boxes indicate pathway-associated proteins detected in this study that were not significantly enriched in either bacterial treatment.

Line 218-221: "Proteins significantly enriched in NJ1-treated (blue) and Hal281-treated (red) nematosomes are mapped onto corresponding pathway components, suggesting differential regulation of lysosomal processing depending on the bacterial isolate. Grey boxes represent proteins detected regarding phagosomes in this study."

Line 405: I am really surprised that polyp length here is not significant – are you sure? Regardless, I think you need to include your stats not just for significant results but also for those analyses that did not produce significance. This could be in a supplemental table or just in the legend or the text, but you should include the t-value and the df, as well as the p-value, for all analyses you did (so not just Fig 4 but also Fig 2, 5, 6).

Response: We thank reviewer for this important suggestion. Statistical reporting has been expanded throughout the revised manuscript to include relevant test statistics, degrees of freedom, and p-values for both significant and non-significant comparisons where applicable. In addition, we removed the polyp size analysis (length and area of polyps) from the revised manuscript, as the measurements were highly influenced by culture conditions and therefore did not allow robust biological interpretation. Instead, all phenotypic analyses were performed using carefully size-matched animals to ensure comparability between experimental groups.

Line 440: The color code in the lower left box of A is almost illegible, even when zoom is increased

Response: Figure readability was improved by increasing font size and contrast in the revised panel.

Line 443: It would be good to discuss some of the strain differences you see in 5B, 2C, etc, somewhere in the paper.

Response: We thank the reviewer for this valuable suggestion. We have now expanded the Discussion to address the observed strain-specific differences between bacterial isolates. In particular, we now discuss that although native Vibrio isolates generally induced lower phagocytotic responses compared to foreign isolates, measurable differences were also detectable among the native and non-native groups. We further integrated recent findings from the squid-Vibrio symbiosis demonstrating that closely related bacterial strains can elicit distinct host responses depending on their evolutionary and ecological background supporting the concept of fine-scale host discrimination in beneficial host-microbe interactions.

Line 521-526: "Interestingly, we also observed differences among the native Vibrio isolates themselves, indicating that nematosomes responses are not only able to distinguish between native and foreign bacteria, but also discriminate at the strain level. Similar strain-specific host responses have recently been described in the squid-Vibrio symbiosis, where closely related bacterial strains triggered distinct host transcriptional and developmental programs depending on their evolutionary and ecological origin."

Very minor revisions:

Line 53: into the phagosome

Lines 82-84: Please rework this sentence for greater clarity.

Line 92: represents

Line 93: with a blue

Line 106: Maybe include, "However, there has not yet been any direct evidence…"

Line 108: Here, we show that nematostomes…

Line 111-112: linked to a phagosomal pathway

Line 117: strong evidence that

Line 127: I think separated might be a better word here than organized

Line 136: I don't think you need the /mL for your OD600 – that is implied.

Line 138: in the dark … bacteria were centrifuged

Line 147: nematostomes were stained

Line 150: mounted on a slide

Line 151: Images were taken…

Line 169, 181, 277: prior to _____

Line 178: I think instead of succession here you mean success?

Lines 182-184: Please rework these two sentences for better clarity.

Line 186: and centrifuged at 5000

Line 186-187: I would either remove "to the bottom of the tube" or change to "to collect in the bottom of the tube"

Line 189: vortexing every 15 min

Line 189-190: were snap-frozen at

Line 197: mixed by pipetting … kept on the shaker

Line 276: …and fasttree2 was used to construct the phylogeny.

Line 277: per sample prior

Line 287: Most of your results section is in past tense, make sure you are consistent (here replace are with were)

Line 326: lysosomal activity

Line 345: host metabolism,

Line 347-349: Proteins belonging to… F-actin are especially increased.

Line 356-358: Please rework this sentence for clarity

Line 379: identified a cJUN

Line 384: As the central hub for ___________, cJUN…

Line 402-403. The sentences that start "F2 with…" and "Additional predicted…" are both fragments – please edit to make complete sentences.

Line 421-422: Maybe "the phagocytosis assay developed above"

Line 428: Hal281 did not result in the activation

Line 436-437: Please rework the clause starting "suggesting even" for clarity

Line 543: However, symbionts also rely

Line 565: don't need both "In addition" and "also", choose one

<u>Response:</u> We thank the reviewer for these careful editorial suggestions. All listed textual corrections and wording improvements have been implemented throughout the revised manuscript.

Response to the Reviewers

We sincerely thank the Editor and the Reviewers for their careful evaluation of our revised manuscript and for their positive feedback. We are grateful that both reviewers consider the manuscript substantially improved and suitable for publication. In response to the remaining comment from Reviewer #1, we have made a minor revision to further clarify the interpretation of the maternal effects observed in the cJUN knockout animals. Our point-by-point responses are provided below.

Reviewer #1

Comment:

The authors have done a very good job with this revision and I consider the manuscript significantly improved and ready for publication. One small point, regarding comment 4.3: given that cJun is expressed in several tissues (not just nematosomes - Fig. 4a) and given that the knockout is not tissue-specific, the authors should use caution in attributing the decreases in offspring fitness to maternal provisioning of nematosomes (Line 266–281 results). There could be other (unmeasured) effects of cJun KO that impacted offspring survival. I have no other concerns – nice job with the revisions!

Response: We thank the reviewer for the positive evaluation of our revised manuscript and for this thoughtful suggestion. We agree that, because the cJUN knockout is not tissue-specific and cJUN is expressed in multiple tissues, we cannot exclude additional cJUN-dependent maternal effects that may contribute to the reduced offspring survival. We have therefore revised the Discussion to clarify that the observed reduction in offspring fitness is consistent with a contribution of maternal nematosome provisioning but cannot be exclusively attributed to reduced maternal transfer of nematosomes. This revised wording more accurately reflects the limitations of the current study while preserving the interpretation supported by our data.

Line 369-375 (Discussion): "In addition to adult polyps, reduced nematosomes abundance was also observed in egg packages derived from cJUN$^{-/-}$ females and was associated with reduced offspring fitness in a mother genotype-dependent manner. These observations are consistent with the hypothesis that maternal provisioning of nematosomes contributes to transgenerational immune protection. However, because cJUN is expressed multiple tissues, we cannot exclude that additional cJUN-dependent maternal effects contribute to the reduced offspring survival."

Reviewer #2

Comment:

You have done a nice job of addressing all the comments raised in the original review. I was happy with the revisions and think this paper is ready to be published.

Response: We sincerely thank the reviewer for the positive assessment of our revised manuscript and for recognizing our efforts to address all previous comments. We appreciate the reviewer's constructive feedback throughout the review process and are pleased that the manuscript is now considered suitable for publication.
